# Transcriptomics in Interferon-*α*-Treated Patients Identifies Inflammation-, Neuroplasticity- and Oxidative Stress-Related Signatures as Predictors and Correlates of Depression

**DOI:** 10.1038/npp.2016.50

**Published:** 2016-04-27

**Authors:** Nilay Hepgul, Annamaria Cattaneo, Kosh Agarwal, Sara Baraldi, Alessandra Borsini, Chiara Bufalino, Daniel M Forton, Valeria Mondelli, Naghmeh Nikkheslat, Nicola Lopizzo, Marco A Riva, Alice Russell, Matthew Hotopf, Carmine M Pariante

**Affiliations:** 1 Department of Psychological Medicine, Institute of Psychiatry, Psychology and Neuroscience, King's College London, London, UK; 2 IRCCS Fatebenefratelli, University of Brescia, Brescia, Italy; 3 Institute of Liver Studies, King's College Hospital, London, UK; 4 Department of Gastroenterology and Hepatology, St George's Hospital, London, UK; 5 Department of Pharmacological and Biomolecular Sciences, University of Milan, Milan, Italy

## Abstract

Owing to the unique opportunity to assess individuals before and after they develop depression within a short timeframe, interferon-*α* (IFN-*α*) treatment for chronic hepatitis C virus (HCV) infection is an ideal model to identify molecular mechanisms relevant to major depression, especially in the context of enhanced inflammation. Fifty-eight patients were assessed prospectively, at baseline and monthly over 24 weeks of IFN-*α* treatment. New-onset cases of depression were determined using the Mini International Neuropsychiatric Interview (MINI). Whole-blood transcriptomic analyses were conducted to investigate the following: (1) baseline gene expression differences associated with future development of IFN-*α*-induced depression, before IFN-*α*, and (2) longitudinal gene expression changes from baseline to weeks 4 or 24 of IFN-*α* treatment, separately in those who did and did not develop depression. Transcriptomics data were analyzed using Partek Genomics Suite (1.4-fold, FDR adjusted *p*⩽0.05) and Ingenuity Pathway Analysis Software. Twenty patients (34%) developed IFN-*α*-induced depression. At baseline, 73 genes were differentially expressed in patients who later developed depression compared with those who did not. After 4 weeks of IFN-*α* treatment, 592 genes were modulated in the whole sample, representing primarily IFN-*α*-responsive genes. Substantially more genes were modulated only in patients who developed depression (*n*=506, compared with *n*=70 in patients who did not), with enrichment in inflammation-, neuroplasticity- and oxidative stress-related pathways. A similar picture was observed at week 24. Our data indicate that patients who develop IFN-*α*-induced depression have an increased biological sensitivity to IFN-*α*, as shown by larger gene expression changes, and specific signatures both as predictors and as correlates.

## Introduction

The development of clinically significant depression during interferon-*α* (IFN-*α*) therapy for chronic hepatitis C virus (HCV) infection is common, with an incidence of up to 45% (Asnis and De La Garza, 2006). Extensive research has been conducted to understand the biological systems involved in the development of IFN-*α*-induced depression and to identify biological predictors associated with an enhanced risk to develop depressive symptoms. However, the molecular mechanisms are still unclear. Owing to the unique opportunity to assess individuals before and after they develop depression within a short period of time (weeks rather than months or years), this model can also crucially identify mechanisms relevant to major depression at large, or at least in the context of increased inflammation. Previous studies have used hypothesis-based approaches, focussed on specific biomarkers (Capuron *et al*, 2003; Lotrich *et al*, 2013; Raison *et al*, 2009). However, IFN-*α* activates many biological systems; therefore, a hypothesis-free approach may identify novel molecular mechanisms that predict or correlate with the development of depression.

Peripherally, IFN-*α* acutely induces the production and release of other innate immune cytokines such as interleukin-6 (IL-6) and tumor necrosis factor-*α* (TNF-*α*) (Raison *et al*, 2008). These cytokines are putatively involved in the depressogenic action of IFN-*α*. Higher serum or plasma levels of these (and other) pro-inflammatory markers have been shown to be associated with an increased risk of major depressive disorder (Dowlati *et al*, 2010). Our own work has shown increased inflammation in the blood of depressed patients when compared with healthy controls together with an association between higher cytokine levels and lack of antidepressant response (Cattaneo *et al*, 2013). Post-mortem gene expression studies show an upregulation of a variety of pro- and anti-inflammatory cytokines in the prefrontal cortex of depressed patients (Shelton *et al*, 2011), thus indicating that peripheral inflammation may correlate with central abnormalities. However, the link between increased peripheral inflammation and IFN-*α*-induced depression is still unclear and studies report mixed results. For example, Wichers *et al* (2006) report a difference in plasma levels of IL-6 between patients who develop IFN-*α*-induced depression and those who do not, but no difference in plasma levels of TNF-*α*. Conversely, Raison *et al* (2008) report increased plasma concentrations of TNF-*α* to be significantly correlated with increased depression scores but no correlation was observed for IL-6.

A consolidated method to investigate the pathogenesis of psychiatric disorders is the use of peripheral blood to measure gene expression (mRNA) levels, which may be considered indicative of gene expression profiles in the brain (Hepgul *et al*, 2013). Several studies have shown that blood cells share >80% of the transcriptome with other tissues, including the brain (Liew *et al*, 2006). A comparison of the transcriptional profiling of 79 human tissues showed that whole blood shares significant gene expression similarities with multiple brain tissues, in particular for genes encoding for neurotransmitter receptors and transporters, stress mediators, cytokines, hormones, and growth factors, all of which are relevant to depression (Sullivan *et al*, 2006). Global gene profiling in IFN-*α*-treated cells has shown a number of IFN-stimulated genes (Wang and Campbell, 2005) such as IFN-induced 15-kDa protein (*ISG15*), ubiquitin-specific proteinase 18 (*USP18*), IFN-induced 10-kDA protein (*IP-10* or chemokine (C-X-C motif) ligand 10 (*CXCL10*)), signal transducers and activators of transcription (*STAT1*), and IFN-induced guanylate-binding protein 3 (*GBP3*), and again with similarities between immune and brain cells (Wang *et al*, 2008). Of specific relevance to the present study, to date only five studies have investigated peripheral blood gene expression changes in the development of IFN-*α*-induced depression. Three of these studies used a candidate gene approach, thus limiting the identification of novel or hitherto unknown mechanisms (Birerdinc *et al*, 2012; Krueger *et al*, 2011; Pawlowski *et al*, 2014), whereas two studies used transcriptomics (Felger *et al*, 2012; Schlaak *et al*, 2012). Felger *et al* (2012) used mRNA from isolated peripheral blood mononuclear cells (PBMCs) of 11 HCV patients before and after 12 weeks of IFN-*α* treatment. They found 252 upregulated and 116 downregulated genes after 12 weeks of IFN-*α* treatment. However, the sample size was small (only four patients developed depressive symptoms) and only two genes were found to be differentially expressed in patients with depression: the 2'-5'-oligoadenylate synthetase 2 (*OAS2*; upregulated) and the high-affinity IgE receptor (*FCER1A*; downregulated). In the other transcriptomics study, blood mRNA was examined 12 h before and 12 h after the first injection of IFN-*α* (Schlaak *et al*, 2012). IFN-*α*-induced depression (after at least 3 months of treatment) was found to be associated with upregulation of 15 genes, suggesting that very early responses to IFN-*α* are related to the development of depression.

In this study, we have used whole-blood transcriptomics to investigate, in a large sample of HCV patients, the following: (1) baseline gene expression differences associated with future development of IFN-*α*-induced depression, before IFN-*α* administration, and (2) longitudinal changes in gene expression from baseline to treatment week 4 (TW4) and TW24 of IFN-*α*, separately in those who did and did not develop depression. In addition, a small number of candidate cytokines were assessed in the plasma of patients at the same time points.

## Materials and methods

### Study Design

This was a prospective cohort study, evaluating patients at baseline and monthly over 24 weeks of IFN-*α* treatment. Blood samples for whole-blood mRNA analysis were collected in PAXgene Blood RNA Tubes (PreAnalytiX, Switzerland) and for plasma cytokine measurement in 2 ml K3EDTA tubes (ThermoFisher Scientific, Massachusetts, USA) using standard protocols, at baseline and at TW4 and TW24. We recruited 58 participants from the outpatient liver departments of three London hospitals: King's College Hospital, Guy's and St Thomas' Hospital, and St George's Hospital. Eligible participants were adults with chronic HCV infection due to commence combination antiviral therapy with IFN-*α* and ribavirin for at least 24 weeks; this comprised weekly subcutaneous IFN-*α* injections (1.5 μg per kg of body weight) and daily ribavirin tablets (800–1400 mg/day orally, in 2 divided doses). Exclusion criteria included the following: age below 18 years, current diagnosis (at baseline) of major depressive disorder, autoimmune disorder, current use of antidepressants, lack of English language, and co-infection with HIV or hepatitis B. Written informed consent was obtained from all participants. The research team did not interfere with the usual clinical practice; when the research team detected depression development during IFN-*α*, this information was communicated to the treating hepatitis clinical team, who in turn decided whether or not to refer to liaison psychiatry services. The study was approved by the King's College Hospital Research Ethics Committee (Ref: 10/H0808/30).

### Questionnaires, Clinical Assessment, and Sample Description

The Mini International Neuropsychiatric Interview (MINI) was administered at baseline, to assess current depression or a previous history of depression, and at follow-up assessments for the detection of new-onset cases of depression. The MINI is a structured diagnostic interview for psychiatric disorders according to the Diagnostic and Statistical Manual of Mental Disorders, 4th Edition and the International Statistical Classification of Diseases and Related Health Problems 10th Revision (Sheehan *et al*, 1998). For the purpose of this study, we only focused on the detection and diagnosis of major depressive episode. In addition, the severity of depressive symptoms was assessed using the Inventory of Depressive Symptomatology (IDS) (Rush *et al*, 1986). IDS scores were significantly higher at TW4 and TW24 when compared with baseline (20.8±1.7 *vs* 11.6±1.5, *p*<0.001 and 22.9±2.0 *vs* 11.6±1.5, *p*<0.001, respectively), whereas there was no significant difference between TW4 and TW24 (20.8±1.7 *vs* 22.9±2.0, *p*=0.2).

The depressed group was defined by a MINI diagnosis of major depressive episode at any time point during the 24 weeks. Four (7%) patients developed depression by TW4 and 20 (34%) patients developed depression by TW24, whereas 38 patients (66%) did not develop depression. Two patients were started on antidepressant treatment after they developed IFN-*α*-induced depression. The socio-demographic and clinical characteristics of the sample are presented in Table 1. Patients who developed depression were significantly more likely to have a previous history of depression, be unemployed, and have higher baseline depression scores. These three variables were interrelated and, indeed, those with a history of depression were more likely to be unemployed and to have higher depression scores at baseline (data not shown). Given this interrelation and the existing evidence for the presence of mood or anxiety symptoms before treatment as a risk factor (Dieperink *et al*, 2003; Lotrich *et al*, 2007), baseline depression scores were used as a covariate in all statistical analyses.

### RNA Isolation and Transcriptomics Analyses

Isolation of total RNA was performed using the PAXgene blood miRNA kit according to the manufacturer's protocol (PreAnalytiX, Hombrechtikon, CHE). RNA quantity and quality were assessed by evaluation of the A260/280 and A260/230 ratios using a Nanodrop spectrophotometer (NanoDrop Technologies, Delaware, USA) and samples kept at −80 °C until processing for whole-genome transcriptomics analyses. Microarray assays were performed following the protocol described in the Affymetrix GeneChip Expression Analysis technical manual (Affymetrix, California, USA). Briefly, 250 ng RNA were used to synthesize cDNA with the Ambion WT Expression Kit (ThermoFisher Scientific), which was then purified, fragmented, labeled, and hybridized onto Human Gene 1.1 ST Array Strips (Affymetrix). The reactions of hybridization, fluidics, and imaging were performed on the Affymetrix GeneAtlas platform (Affymetrix) instrument according to the manufacturer's protocol. Validation of transcriptomics was performed using real-time PCR (full details in Supplementary Materials). Correlation between the Affymetrix and real-time values was 0.99 (data presented in Supplementary Table S1).

### Plasma Cytokine Measurement

Blood samples were collected in 2 ml K3EDTA tubes (ThermoFisher Scientific). On arrival to the laboratory, samples were centrifuged at 1500 *g* for 15 min at room temperature and plasma were removed and frozen at −80 °C until processing for cytokine measurement. All candidate proteins were measured using Magnetic Luminex Performance Multiplex Assay (R&D Systems, Minneapolis, USA), using the customized 7-plex Human High Sensitivity Cytokine Premixed kit (R&D, FCSTM14). From the cytokines available for ‘high-sensitivity' measurement, we chose five cytokines known to be stimulated by IFN-*α* (IL-1*β*, IL-2, IL-6, IFN-*γ*, and TNF-*α* (Taylor and Grossberg, 1998)) and two that are inhibited (IL-7 (Su *et al*, 1997) and IL-17A (Cui *et al*, 2014)). All samples were assayed according to the manufacturer's protocol and the results analyzed using SoftMax Pro V4.8 (full details in Supplementary Materials). IFN-*γ* levels were below detection limit for most samples and therefore were not analyzed further.

### Statistical and Bioinformatic Analyses

Data were analysed using IBM SPSS V20. Continuous variables are presented as mean±SEM. Differences in clinical and socio-demographic variables between patients who developed IFN-*α*-induced depression and those who did not were analyzed using independent samples *t*-tests. Changes in depression scores between time points were analyzed using paired samples *t*-tests. A repeated-measures ANCOVA (with baseline IDS scores as a covariate) was performed, to test for differences in plasma cytokine levels as an effect of IFN-*α* treatment (time effect) and in relation to depression development (group effect). For gene expression data, CEL files were imported into Partek Genomics Suite V6.6 for data visualization and quality control. In summary (full details in Supplementary Materials), background correction was conducted using Robust Multi-strip Average (Irizarry *et al*, 2003) and Quantile Normalization (Bolstad *et al*, 2003) was used to normalize the distribution of probe intensities among different microarray strips. Summarization was conducted using a linear median polish algorithm (Tukey, 1977) to integrate probe intensities and compute expression levels for each gene transcript. To assess the effect of IFN-*α* treatment, a multiple linear contrast over time was performed and gene lists obtained by applying cutoffs of both *p*-value (FDR corrected) of ⩽0.05 and a minimum absolute fold change of 1.4. Ingenuity Pathway Analysis Software was used to identify regulation of molecular signaling pathways.

## Results

### Baseline Gene Expression Differences Predicting the Development of IFN-*α*-Induced Depression

**Figure 1:**
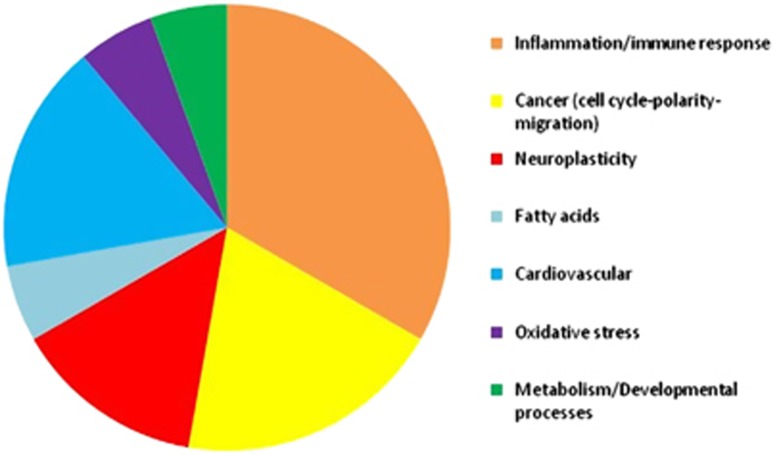
Role classification of pathways differentially modulated at baseline between patients who develop depression compared with those who do not.

We first examined differences in gene expression at baseline (before starting IFN-*α*) and compared the profile of patients who subsequently did and who did not develop IFN-*α*-induced depression. Using the above-mentioned cutoff criteria and baseline depression scores as a covariate, we found 73 differentially modulated genes (see Supplementary Table S2). Pathway analysis of these 73 genes identified 24 pathways including inflammation-, neuroplasticity- and oxidative stress-related pathways, such as IL-1 signaling, NRF2-mediated oxidative stress response, and axonal guidance signaling. These pathways are presented in Figure 1 and Table 2. In addition, we also compared plasma cytokine levels at baseline; however, there were no significant differences between the two groups (all *p*-values >0.2; see Supplementary Table S3).

### Genes Modulated by IFN-*α* at TW4 in the Whole Sample and in Relation to Development of IFN-*α*-Induced Depression

**Figure 2:**
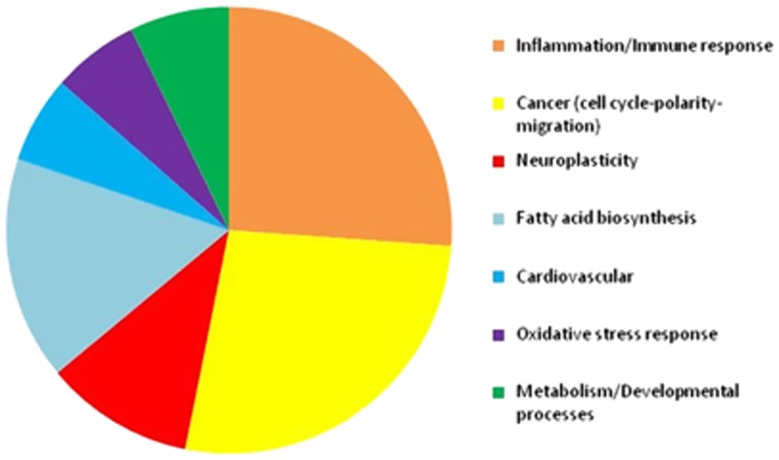
Role classification of pathways differentially modulated at treatment week 4 (TW4) specifically in patients who develop depression.

We identified genes modulated by IFN-*α*, by comparing the expression profile of the sample at TW4 with the profile at baseline. IFN-*α* modulated 592 genes, including well-known IFN-*α* targets such as IFN-*α*-inducible protein 27 (*IFI27*, FC=+32.05), IFN-induced protein 44-like (*IFI44L*, FC=+11.59), *USP18* (FC=+4.39), ISG15 ubiquitin-like modifier (*ISG15*, FC=+2.70), and *CXCL10* (FC=+1.84). Subsequently, we investigated genes modulated by IFN-*α* separately in patients who developed depression (*n*=20) and those who did not (*n*=38). We found 506 genes modulated only in patients who developed depression (see Supplementary Table S4) and 70 genes modulated only in patients who did not develop depression (see Supplementary Table S5). We focussed our further analyses on the 506 genes specifically modulated in patients who developed depression. We found 224 upregulated and 284 downregulated genes, including genes previously found to be associated with depression such as *CXCL10* (FC=+2.13) and insulin-like growth factor 2 mRNA binding protein 2 (*IGF2BP2*, FC=+1.88). Pathway analysis of these 506 genes identified 65 pathways including those related to inflammation (IL-1, IL-6, and IL-8 signaling, glucocorticoid receptor (GR) signaling, triggering receptor expressed on myeloid cells 1 signaling, and nuclear factor-*κ*B cells (NF-*κ*B) signaling), neuroplasticity (extracellular signal-regulated kinase 5 (ERK5) signaling and axonal guidance signaling), and oxidative stress (NRF2-mediated oxidative stress response, p53 signaling, and production of nitric oxide and reactive oxygen species in macrophages). These pathways are presented in Figure 2 and Table 3.

Finally, we conducted a two-way repeated-measure ANCOVA (with baseline depression scores as a covariate) to investigate changes in plasma cytokine levels between TW4 and baseline, and also in relation with depression development. We found a significant effect of IFN-*α* (time effect) for decreasing IL-1*β* (*p*=0.043) and increasing IL-6 (*p*=0.013), IL-17A (*p*=0.022), and TNF-*α* (*p*<0.001), but no effect of depressive status (group effect, all *p*-values >0.3) nor time by group effect (all *p*-values >0.2) (see Supplementary Table S3).

### Genes Modulated by IFN-*α* at Week 24 in the Whole Sample and in Relation to Development of IFN-*α*-Induced Depression

In order to assess whether IFN-*α*-modulated changes persisted further down the treatment course, we compared the gene expression profile of patients at TW24 with the profile at baseline. Similar to TW4, modulation of several IFN-*α* target genes were observed in the whole sample, including *IFI27* (FC=+34.20), *IFI44L* (FC=+11.78), *USP18* (FC=+4.72), *ISG15* (FC=+2.80), and *CXCL10* (FC=+2.52). In addition, we observed modulation of other known IFN-*α* target genes, which we did not see at TW4, such as *GBP3* (FC=+1.45) and *STAT1*, 91 kDa (FC=+1.45). We found 285 genes modulated only in patients who developed depression (see Supplementary Table S6) and 121 genes modulated only in patients who did not develop depression (see Supplementary Table S7). We focussed our further analyses on the 285 genes that were modulated in patients who developed depression. These included 215 upregulated and 112 downregulated genes. Pathway analysis of these 285 genes identified 20 pathways. Interestingly, as indicated in Table 3, eight pathways were modulated at both TW4 and TW24, including ERK5 signaling, stress-activated protein kinase/Jun N-terminal kinase (SAPK/JNK) signaling, and the glutathione redox reactions I pathway. The full list of 20 pathways and their role classifications can be seen in Supplementary Table S8 and Supplementary Figure S9, respectively.

Again, we conducted a two-way repeated-measure ANCOVA (with baseline depression scores as a covariate), to investigate changes in plasma cytokine levels between TW24 and baseline, and in relation with depression development. We found a significant effect of IFN-*α* (time effect) for increasing IL-6 (*p*=0.012), IL-17A (*p*=0.008), and TNF-*α* (*p*<0.001), but again no effect of depressive status (group effect, all *p*-values >0.4) nor time by group interaction (all *p*-values >0.1) (see Supplementary Table S3).

## Discussion

To our knowledge, this is the first study to use a peripheral blood transcriptomics approach to identify both predictors of future development of IFN-*α*-induced depression and biological pathways associated with the development of depression. Already before IFN-*α*, patients who later develop depression show significant differences in the expression of several pathways compared with those who do not; in addition, by TW4 they show specific longitudinal changes in similar and additional pathways, related to inflammation, neuroplasticity, and oxidative stress. We replicate a number of previously identified IFN-responsive genes. For example, Wang *et al* (2008) listed *ISG15*, *USP18*, *IP*-*10*/*CXCL10*, *STAT1*, and IFN-induced *GBP3* as some of the most highly expressed IFN responsive genes and we show changes in all of these genes following IFN-*α* administration. In addition, we replicate IFN-*α*-stimulated genes found in cultured neurons including *IRF7*, *PNPT1*, and *IFIH1* (Wang and Campbell, 2005). Finally, we replicate 100 genes (35%) and 5 pathways, shown to be responsive to IFN-*α* by Felger *et al* (2012). These consistencies strengthen the validity of our findings.

Among the most significant differentially expressed genes at baseline (predictors), we find upregulation of ubiquitin-fold modifier 1 *(UFM1)* and eukaryotic translation initiation factor 4B *(EIF4B)* in patients who later develop IFN-*α*-induced depression. The exact functions of UFM1 are poorly understood; however, it has been shown to be involved in heart disease (Azfer *et al*, 2006) and diabetes (Lemaire *et al*, 2011) (conditions that present frequent comorbidity with depression), as well as schizophrenia (Rubio *et al*, 2013). EIF4B is a downstream component of the mammalian target of rapamycin (mTOR) signaling pathway. There is abundant evidence linking mTOR signaling to synaptic plasticity, memory, and psychiatric disorders (Hoeffer and Klann, 2010). Although we see increased expression of *EIF4B* in our patients who subsequently develop IFN-*α*-induced depression, Jernigan *et al* (2011) report significant reductions in *EIF4B* (and mTOR) expression in the prefrontal cortex of depressed subjects. The sample used by Jernigan *et al* (2011) were on overage more than 10 years older and were predominantly suicide victims, which may account for these discrepant results. Also at baseline, we identified 24 pathways, some related to inflammation (IL-1 and CCR3 signaling), neuroplasticity (axonal guidance and netrin signaling), and oxidative stress (NRF2-mediated oxidative stress response and glutathione redox reactions I signaling). Some of these pathways were also significantly regulated longitudinally in patients who develop depression (see below).

Following 4 weeks of IFN-*α* treatment, we find that the number of modulated genes is more than seven times larger in patients who develop depression than in patients who do not, suggesting that patients who develop IFN-*α*-induced depression have an increased biological sensitivity to IFN-*α*. Of note, only four patients were depressed at TW4, while the majority became depressed between week 4 and 12 (*n*=11) or between week 12 and 24 (*n*=5); therefore, most patients classified as depressed in these analyses were on a trajectory to develop depression but had not yet developed it. As such, gene expression changes at TW4 could be conceptualized as early biological changes associated with future depression development, rather than as a consequence of a depressive status. In this regard, our findings are consistent with those of Capuron *et al* (2003) and Schlaak *et al* (2012), who have demonstrated that cortisol responses and, respectively, gene expression changes, after the first injection of IFN-*α*, predict the development of depression after 12 weeks of treatment.

Our findings confirm and extend previous gene expression studies in patients with IFN-*α*-induced depression. Felger *et al* (2012) used a different transcriptomics approach (isolated PBMCs and cross-sectional comparison at week 12). In relation to depression, they found two genes differentially regulated in those who develop depression: *OAS2* (upregulated) and *FCER1A* (downregulated). We also found that these two genes were regulated in the same direction by IFN-*α* and found the change in *OAS2* to be larger in patients who developed depression than in those who did not (FC=+4.13 *vs* FC=+3.06, respectively). Krueger *et al* (2011), Birerdinc *et al* (2012), and Pawlowski *et al* (2014) all used a candidate gene approach focused on key immune genes only and in smaller samples. They all found a variety of cytokines or cytokine target genes differentially modulated in depressed patients. Although we do not directly replicate any of these specific genes, we find an involvement of several inflammatory pathways including IL-6, IL-1, IL-8, and NF-*κ*B. Finally, in their transcriptomics study, Schlaak *et al* (2012) identified 15 genes regulated 12 h after the first injection of IFN-*α* in 11 patients who developed depression (in comparison with 11 patients who did not develop depression). Although we do not replicate these genes exactly, we find five genes belonging to the same family of genes (guanylate-binding proteins, glutaredoxins, proteasome subunit-*β*type, TNF superfamily members, and zinc finger proteins).

Our pathway analysis data show that patients who develop depression also exhibit modulation in oxidative stress pathways such as NRF2-mediated oxidative stress response, the glutathione redox reactions I, and production of nitric oxide and reactive oxygen species in macrophages pathways. Glutathione has an important role in detoxifying various reactive oxygen species, which are known to induce oxidative stress (Franco *et al*, 2007). This finding is consistent with the evidence that depression is associated with increased oxidative and nitrosative stress, both in humans and in animal models (Bakunina *et al*, 2015). We also demonstrate the involvement of neuroplasticity pathways such as ERK5 signaling. Indeed, major depressive disorder may involve a reduced ability of neuronal systems to show adaptive plasticity, especially under stress conditions. There is a wealth of evidence, indicating reductions in various neurotrophic factors in the plasma and serum, and more recently also in the gene expression, of depressed patients (Cattaneo *et al*, 2010; Dwivedi *et al*, 2005; Otsuki *et al*, 2008). ERK5 is a member of the mitogen-activated protein kinase (MAPK) family that includes ERK1/2, p38, and JNK, and participates in the neuronal modulation of depression (Todorovic *et al*, 2009). Indeed, the modulation of the ERK5 signaling pathway is accompanied by alterations in the SAPK/JNK signaling pathway in our patients who develop IFN-*α*-induced depression. Although ERK5 has not previously been implicated in the development of IFN-*α*-induced depression, activation of p38 MAPK in peripheral blood lymphocytes following the initial injection of IFN-*α* has been shown to be implicated (Felger *et al*, 2011). One mechanism through which p38 MAPK can be depressogenic is by increasing the activity and expression of the serotonin transporter and by inhibiting GR function (Wang *et al*, 2004). This is in keeping with our finding for a modulation of the GR signaling pathway, specifically in our depressed group. Reduced GR function (glucocorticoid resistance) coupled with high levels of cortisol is an indicator of hypothalamic–pituitary–adrenal axis dysfunction and is one of the most replicated biological findings in depression, demonstrated also in gene expression studies (Matsubara *et al*, 2006). Glucocorticoid resistance has also been hypothesized to underlie the enhanced inflammation described in major depression (Pariante and Lightman, 2008).

We also measured a small number of candidate cytokines in the plasma of our patients. Although we find a significant effect of IFN-*α* treatment in the whole sample, we do not detect any differences between patients who developed IFN-*α*-induced depression and those who did not. Indeed, previous studies examining peripheral inflammation and IFN-*α*-induced depression have produced inconsistent results. For example, as mentioned previously, Wichers *et al* (2006) report a difference in plasma IL-6 levels between patients who develop IFN-*α*-induced depression and those who do not, but no difference in plasma TNF-*α* levels. Conversely, Raison *et al* (2008) report increased plasma TNF-*α* concentrations to be significantly correlated with increased depression scores but no correlation was observed for IL-6. One study found no effect of IFN-*α* itself on plasma levels of key cytokines but instead found an increase in CSF concentrations (Raison *et al*, 2009). Although we found an effect of IFN-*α* on increasing IL-6, IL-17A, and TNF-*α*, in general these studies and our findings confirm the notion that peripheral cytokines are not an accurate biomarker with reference to the effects of IFN-*α*. As such, we believe investigating gene expression is a more accurate and reliable method, and indeed none of the previous gene expression studies conducted in IFN-*α*-induced depression measured peripheral levels of cytokines.

Our findings are both biologically and clinically relevant. Studying such a clearly defined patient group at ‘very high risk' of developing depression within a few weeks is a useful model to understand the pathogenesis of depression, especially depression in the context of enhanced inflammation, as it has been described after exposures to childhood trauma (Danese *et al*, 2008) or due to genetic predisposition (Bufalino *et al*, 2013). Of note, a recent transcriptomics study identified similar gene expression changes (in IL-6 and NF-*κ*B signaling pathways) as correlates of antidepressant response to the TNF antagonist, infliximab, supporting the notion that our findings are relevant for this broader context (Mehta *et al*, 2013). There is also evidence that increased inflammation is associated with the lack of antidepressant response (Cattaneo *et al*, 2013; Guilloux *et al*, 2015), and therefore it is possible that the genes identified in our study are relevant to antidepressant response. Of note, in the context of HCV infection, biological predictors of IFN-*α*-induced depression can still have a role in clinical practice, even in light of emerging IFN-*α*-free treatment regimens. At present, there are several drugs licenced for use in HCV infection without IFN-*α* administration (Ryder, 2015); however, these treatments are not yet readily available for all viral genotypes and are highly expensive. As such, IFN-*α*-induced depression remains a clinical burden for some populations.

There are some limitations of this study. First, we acknowledge there are tissue-specific differences in gene expression patterns and by using whole-blood mRNA we cannot understand which cell types are responsible for the changes we observe and how these may reflect changes in other tissues such as the brain. However, studies have shown a satisfactory degree of correlation between gene expression in the blood and in the brain (Cai *et al*, 2010; Liew *et al*, 2006; Sullivan *et al*, 2006), and indeed, as mentioned above, we replicate (in peripheral blood) the pattern of IFN-responsive genes previously described in IFN-*α*-stimulated neurons (Wang and Campbell, 2005). Therefore, we believe that this issue does not detract from the impact our findings. Second, all of the patients received combination therapy with IFN-*α* and the antiviral agent ribavirin. Furthermore, although the use of antidepressants was an exclusion criterion at baseline, a very small number of depressed patients were prescribed antidepressants during IFN-*α* (*n*=2). As such, there may be some transcriptional and behavioral changes that could have been influenced by these pharmacological agents.

In conclusion, this study provides several lines of evidence for the possible molecular mechanisms involved in the impact of IFN-*α* on behavior. Beyond IFN-*α* treatment, the identified transcriptomics signatures could be used as biomarkers for the identification of individuals at risk of developing depression, especially in the context of high inflammation due to stress, physical illness, or genetic make-up, or to generate molecular targets for the discovery of new therapeutics in depression.

## Funding and disclosure

Professor Pariante and Dr Mondelli have received research funding from Johnson & Johnson as part of a program of research on depression and inflammation. In addition, Professor Pariante and Dr Mondelli have received research funding from the Medical Research Council (UK) and the Wellcome Trust for research on depression and inflammation as part of two large consortia that also include Johnson & Johnson, GSK, and Lundbeck. Dr Agarwal has received research funding from Bristol-Myers Squibb and Gilead, as well as consulting fees from Achillon, AbbVie, Astellas, Boehringer Ingelheim, Bristol-Myers Squibb, Gilead, Janssen, Merck, and Novartis. Dr Forton has received consulting fees from Merck, Boehringer Ingelheim, Roche, Janssen, AbbVie, BMS, and Gilead. Dr Hepgul, Dr Cattaneo, Dr Baraldi, Dr Bufalino, Ms Borsini, Professor Hotopf, Ms Russell, Mr Lopizzo, and Professor Riva all declare no conflict of interest.

## Figures and Tables

**Table 1:** Socio-Demographic and Clinical Characteristics of the Sample

	**Whole sample** ***n*=58**	**Depressed patients** ***n*=20**	**Non-depressed patients** ***n*=38**	
*Age (years)*				
Mean±SEM	44.7±1.6	42.4±2.5	45.9±2.0	*t*=1.1, df=56, *p*=0.3

*Gender*				
Male	45 (78%)	15 (75%)	30 (79%)	*x*^2^=0.12, *p*=0.5

*Ethnicity*				
White British	27 (47%)	8 (40%)	19 (50%)	*x*^2^=0.53, *p*=0.3
Other	31 (53%)	12 (60%)	19 (50%)	

*Education level*				
University/A level	23 (41%)	8 (40%)	15 (42%)	*x*^2^=0.01, *p*=0.6
GCSEs/no qualifications	33 (59%)	12 (60%)	21 (58%)	

*Employment*				
Full-time	32 (55%)	7 (35%)	25 (66%)	*x*^2^=5.02, ***p*****<0.05**
Unemployed	26 (45%)	13 (65%)	13 (34%)	

*Relationship status*				
Single	27 (47%)	8 (40%)	19 (50%)	*x*^2^=0.53, *p*=0.3
Married/cohabiting	31 (53%)	12 (60%)	19 (50%)	

History of depression	19 (33%)	10 (50%)	9 (24%)	*x*^2^=4.12, ***p*****<0.05**
Family history of psychiatric illness	14 (31%)	5 (36%)	9 (29%)	*x*^2^=0.20, *p*=0.5
Baseline depression scores	11.6±1.5	16.1±3.0	9.2±1.6	*t*=−2.3, df=56, ***p*****<0.05**
TW4 depression scores	20.8±1.7	28.6±2.5	16.6±1.9	*t*=−3.8, df=56, ***p*****<0.001**
TW24 depression scores	22.9±2.0	37.1±3.3	15.5±1.6	*t*=−6.7, df=56, ***p*****<0.001**

*Alcohol use*				
Mean±SEM	4.2±0.9	5.3±2.1	3.7±0.8	*t*=−0.9, df=54, *p*=0.4

*HCV genotype*				
1 and 4	13 (22%)	6 (30%)	7 (18%)	*x*^2^=1.01, *p*=0.2
2 and 3	45 (78%)	14 (70%)	31 (82%)	

*HCV viral load (million)*				
Mean±SEM	2.2±2.8	2.1±0.7	2.2±0.5	*t*=0.2, df=54, *p*=0.8

*Fibroscan scores (kpa)*				
Mean±SEM	9.9±1.2	11.1±2.7	9.3±1.3	*t*=−0.7, df=44, *p*=0.5

Numbers in bold indicate significant results.

**Table 2:** Pathways Differentially Modulated at Baseline Between Patients Who Develop Depression Compared With Those Who Do Not (*p*⩽0.05)

**Pathway**	**Molecules**
Ephrin B signaling	GNAT2, NCK2, ROCK1, GNG2
Coagulation system	A2M, PROS1, SERPIND1
G alpha q signaling	RGS18, PPP3R1, ROCK1, GNG2
Ephrin receptor signaling	GNAT2, NCK2, ROCK1, GNG2
NRF2-mediated oxidative stress response	DNAJC8, DNAJA2, GSTM3, GPX2
Cardiac hypertrophy signaling	PPP3R1, GNAT2, ROCK1, GNG2
CCR3 signaling in eosinophils	PLA2G10, ROCK1, GNG2
Netrin signaling	PPP3R1, NCK2
Axonal guidance signaling	PPP3R1, GNAT2, NCK2, ROCK1, GNG2
CXCR4 signaling	GNAT2, ROCK1, GNG2
Actin nucleation by ARP-WASP complex	NCK2, ROCK1
Role of NFAT in regulation of the immune response	PPP3R1, GNAT2, GNG2
RhoGDI signaling	GNAT2, ROCK1, GNG2
Thrombin signaling	GNAT2, ROCK1, GNG2
Integrin signaling	NCK2, ROCK1, LIMS1
G Beta gamma signaling	GNAT2, GNG2
Signalling by Rho family GTPases	GNAT2, ROCK1, GNG2
IL-1 signaling	GNAT2, GNG2
Phospholipase C signaling	PPP3R1, PLA2G10, GNG2
fMLP signaling in neutrophils	PPP3R1, GNG2
Androgen signalling	GNAT2, GNG2
Extrinsic prothrombin activation pathway	PROS1
Glutathione redox reactions I	GPX2
Relaxin signaling	GNAT2, GNG2

**Table 3:** Pathways Differentially Modulated at TW4 Specifically in Patients Who Develop Depression (*p*⩽0.05)

**Pathway**	**Molecules**
Aryl hydrocarbon receptor signaling	FOS, NCOR2, HSPB1, IL1B, ALDH1A1, NQO2, ALDH5A1, NFIA, MGST1, TFDP1, MGST3, NRIP1
IL-6 signaling	FOS, A2M, HSPB1, IL1RN, IL1B, TNFAIP6, CD14, IL6R, AKT2, IL6ST
ERK5 signaling^a^	FOS, SGK1, GNA12, WNK1, GAB1, MAP3K3, IL6ST
Pentose phosphate pathway^a^	TKT, PGD, TALDO1
NRF2-mediated oxidative stress response	FOS, DNAJA4, DNAJC8, DNAJC6, GCLC, ABCC4, DNAJA2, DNAJC15, NQO2, MGST1, MGST3
Phenylethylamine degradation I^a^	ALDH2, AOC3
NF-*κ*B signaling	BMPR2, TNFSF13B, IL1RN, IL1B, AZI2, TLR6, PELI1, AKT2, MAP3K3, PLCG2
LXR/RXR activation	NCOR2, S100A8, IL1RN, IL1B, MMP9, ORM1, CD14, PTGS2
Ephrin B signaling	ACP1, EPHB4, GNAT2, GNA12, ROCK1, GNG2
Production of nitric oxide and reactive oxygen species in macrophages	FOS, IFNGR1, NCF4, S100A8, ORM1, NCF1, SIRPA, AKT2, MAP3K3, PLCG2
IL-8 signaling	FOS, MMP9, CXCR2, PTGS2, CXCR1, GNA12, ROCK1, BCL2L1, GNG2, AKT2
Phosphatidylglycerol biosynthesis II (non-plastidic)	ABHD5, AGPAT9, PGS1
GR signaling	KAT2B, NCOR2, IL1B, PPP3CA, AKT2, FOS, A2M, IL1RN, DUSP1, SGK1, PTGS2, BCL2L1, NRIP1
Pentose phosphate pathway (non-oxidative branch)^a^	TKT, TALDO1
Rapoport–Luebering glycolytic shunt	BPGM, MINPP1
Glutathione redox reactions I^a^	CLIC2, MGST1, MGST3
Fatty acid alpha oxidation	ALDH1A1, PTGS2, ALDH2
Pancreatic adenocarcinoma signaling^a^	NOTCH1, MMP9, PTGS2, E2F2, BCL2L1, TFDP1, AKT2
RAR activation	FOS, KAT2B, NCOR2, RPL7A, PML, DUSP1, ALDH1A1, AKT2, NRIP1, PRKAR1A
Superpathway of inositol phosphate compounds	PPP1R8, DUSP1, PPTC7, ACP1, PPP4R1, SIRPA, PPP3CA, MINPP1, INPP5A, PLCG2
Pyrimidine deoxyribonucleotides *de novo* biosynthesis I	AK5, NME4, RRM2B
Role of macrophages, fibroblasts and endothelial cells in rheumatoid arthritis	TNFSF13B, F2RL1, IL1B, IL6R, PPP3CA, AKT2, IL6ST, PLCG2, FOS, IL1RN, C5AR1, ROCK1, TLR6
G beta gamma signaling	GNAT2, GNA12, GNG2, AKT2, PLCG2, PRKAR1A
Eicosanoid signaling	CYSLTR2, DPEP2, PTGS2, FPR2, TBXAS1
Colorectal cancer metastasis signaling	FOS, IFNGR1, MMP9, PTGS2, IL6R, BCL2L1, TLR6, GNG2, AKT2, IL6ST, PRKAR1A
D-myo-inositol-5-phosphate metabolism	PPP1R8, DUSP1, PPTC7, ACP1, PPP4R1, SIRPA, PPP3CA, PLCG2
Granulocyte adhesion and diapedesis	HSPB1, IL1RN, IL1B, MMP9, CXCL10, C5AR1, CXCR2, FPR2, HRH2
Superpathway of D-myo-inositol (1,4,5)-trisphosphate metabolism	IMPA2, MINPP1, INPP5A
Prostanoid biosynthesis	PTGS2, TBXAS1
PPAR signaling	FOS, NCOR2, IL1RN, IL1B, PTGS2, NRIP1
EIF2 signaling^a^	RPL5, RPL7A, RPL13A, EIF4A1, RPL41, RPS2, AKT2, RPS15, AGO4
p53 signaling	KAT2B, PML, BCL2L1, AKT2, RRM2B, PMAIP1
Agranulocyte adhesion and diapedesis	IL1RN, IL1B, MMP9, CXCL10, C5AR1, CXCR2, CXCR1, AOC3, MYH9
Toll-like receptor signaling	FOS, IL1RN, IL1B, CD14, TLR6
Cholecystokinin/gastrin-mediated signaling	FOS, IL1RN, IL1B, PTGS2, GNA12, ROCK1
D-myo-inositol (1,4,5,6)-tetrakisphosphate biosynthesis	PPP1R8, DUSP1, PPTC7, ACP1, PPP4R1, SIRPA, PPP3CA
D-myo-inositol (3,4,5,6)-tetrakisphosphate biosynthesis	PPP1R8, DUSP1, PPTC7, ACP1, PPP4R1, SIRPA, PPP3CA
Cardiac hypertrophy signaling	HSPB1, GNAT2, GNA12, IL6R, ROCK1, GNG2, PPP3CA, MAP3K3, PLCG2, PRKAR1A
Sorbitol degradation I	SORD
TREM1 signaling	NLRP12, IL1B, TLR6, AKT2, PLCG2
Pyrimidine ribonucleotides interconversion	ENTPD1, AK5, NME4
Axonal guidance signaling	MMP9, EPHB4, ADAM8, GNAT2, ARHGEF12, GNA12, TUBG1,PLXNC1, ADAM19, PPP3CA, AKT2, PLCG2, PRKAR1A, TUBA1A, ROCK1, GNG2
HGF signaling	FOS, PTGS2, GAB1, AKT2, MAP3K3, PLCG2
Molecular mechanisms of cancer	NOTCH1, GNAT2, ARHGEF12, GNA12, GAB1, AKT2, PRKAR1A, FOS, RALB, BMPR2, E2F2, BCL2L1, TFDP1, PMAIP1
Relaxin signaling	FOS, MMP9, GNAT2, GNA12, GNG2, AKT2, PRKAR1A
Pyrimidine ribonucleotides *de novo* biosynthesis	ENTPD1, AK5, NME4
Acute phase response signaling	FOS, A2M, IL1RN,I L1B, ORM1, IL6R, AKT2, IL6ST
Role of NFAT in regulation of the immune response	FOS, SYK, GNAT2, GNA12, GNG2, PPP3CA, AKT2, PLCG2
Xenobiotic metabolism signaling	NCOR2, GCLC, IL1B, ALDH1A1, UGT2B7, NQO2, ALDH5A1, MGST1, MAP3K3, MGST3, NRIP1
3-Phosphoinositide degradation	PPP1R8, DUSP1, PPTC7, ACP1, PPP4R1, SIRPA, PPP3CA
Triacylglycerol biosynthesis	ABHD5, AGPAT9, LPPR2
Communication between innate and adaptive immune cells	TNFSF13B, IL1RN, IL1B, CXCL10, TLR6
Cardiolipin biosynthesis II	PGS1
GM-CSF signaling	CSF2RA, BCL2L1, PPP3CA, AKT2
G alpha i signaling	RALB, CXCR2, FPR2, P2RY14, GNG2, PRKAR1A
Aldosterone signaling in epithelial cells	HSPB1, DNAJC8, DNAJC6, DUSP1, SGK1, DNAJC15, PLCG2
CDP-diacylglycerol biosynthesis I	ABHD5, AGPAT9
Histamine degradation	ALDH1A1, ALDH2
IL-1 signaling	FOS, GNAT2, GNA12, GNG2, PRKAR1A
RhoA signaling	LPAR6,ARHGEF12, GNA12, ROCK1, CDC42EP2, ARHGAP9
Cell cycle: G1/S checkpoint regulation	RPL5, E2F2, NRG1, TFDP1
Salvage pathways of pyrimidine ribonucleotides	SGK1, AK5, CDK8, NME4, AKT2
Atherosclerosis signaling	S100A8, TNFRSF14, IL1RN, IL1B, MMP9, ORM1
Docosahexaenoic acid (DHA) signaling	IL1B, BCL2L1, AKT2
SAPK/JNK signaling^a^	MAP4K5, GNA12, GNG2, GAB1, MAP3K3
Aryl hydrocarbon receptor signaling	FOS, NCOR2, HSPB1, IL1B, ALDH1A1, NQO2, ALDH5A1, NFIA, MGST1, TFDP1, MGST3, NRIP1

a Pathways which were also modulated at TW24.
