# Supplementary material for: Transcriptomics in Interferon-α-Treated Patients Identifies Inflammation-, Neuroplasticity- and Oxidative Stress-Related Signatures as Predictors and Correlates of Depression
Source: Neuropsychopharmacology. 2016 Apr 27;41(10):2502–11. doi: 10.1038/npp.2016.50 (PMC4983179; doi:10.1038/npp.2016.50)
Supplement: Supplementary Information [file npp201650x1.docx]

***Supplemental Material***

**Transcriptomics in interferon-alpha treated patients identifies inflammation-, neuroplasticity- and**

**oxidative stress-related signatures as predictors and correlates of depression**

**(Running title: Transcriptomics in interferon-α-induced depression)**

Nilay Hepgul PhD^1^, Annamaria Cattaneo PhD^1,2^, Kosh Agarwal MD^3^, Sara Baraldi MD^1^, Alessandra Borsini BSc^1^, Chiara Bufalino MD^1^, Daniel M. Forton MD, FRCP^4^, Valeria Mondelli PhD^1^, Naghmeh Nikkheslat PhD^1^, Nicola Lopizzo MSc^2^, Marco A. Riva PhD^5^, Alice Russell BSc^1^, Matthew Hotopf MD, FRCP^1^, Carmine M. Pariante MD. MRCPsych^1^

^1^Department of Psychological Medicine, Institute of Psychiatry, Psychology & Neuroscience, King’s College London, London, UK. ^2^IRCCS Fatebenefratelli, University of Brescia, Brescia, Italy. ^3^Institute of Liver Studies, King’s College Hospital, London, UK. ^4^Department of Gastroenterology & Hepatology, St. George’s Hospital, London, UK. ^5^Department of Pharmacological and Biomolecular Sciences, University of Milan, Milan, Italy.

*Validation of transcriptomics through Real Time PCR*

Nine genes were measured using Real Time PCR analyses as a validation step: 3 genes differentially modulated at baseline between patients who develop depression and those who do not and 6 genes modulated at week 4 in the whole sample. Whole blood mRNA gene expression levels were analysed by TaqMan qRT-PCR instrument (CFX384 real time system, Bio-Rad, California, USA) using the iScriptTM one-step RT-PCR kit for probes (Bio-Rad, California, USA). Samples were run in 384-well formats in triplicate as multiplexed reactions, and each target gene normalized to the expression of three housekeeping genes: glyceraldehyde 3-phosphate dehydrogenase (GAPDH), beta-actin (b-actin), and beta-2-micro-globulin (B2M). Probe and primer sequences for all target and housekeeping genes were purchased from Life Technologies (ThermoFisher Scientific, Massachusetts, USA). For each sample, 30ng of RNA were added to the Real Time PCR Mix. Thermal cycling was initiated with an incubation at 50°C for 10 minutes (RNA retrotranscription), then 95°C for 5 minutes (TaqMan polymerase activation). After this, 39 cycles of PCR were performed with each cycle consisting of heating samples at 95°C for 10 seconds to enable the melting process, then at 60°C for 30 seconds for annealing and extension. Relative target gene expression was calculated according to the 2(-Delta Delta C(T)) method. Correlation between the Affymetrix and Real Time values was 0.99 (data presented in **Supplemental Table S1)**.

*Plasma cytokine measurement*

Blood samples were collected in 2ml K3EDTA tubes (ThermoFisher Scientific, Massachusetts, USA). On arrival to the laboratory, the samples were centrifuged at 1500g for 15 minutes at room temperature, and then plasma removed and frozen at -80°C until processing for cytokine measurement. All candidate proteins were measured utilizing Magnetic Luminex® Performance Multiplex Assay (R&D Systems Inc., Minneapolis, USA), which performs a solid phase approach to analyze multiple analytes in a single sample. The customised 7-plex Human High Sensitivity Cytokine Premixed kit (R&D, FCSTM14) was used to determine the plasma levels of: interleukin-1 beta (IL-1β), interleukin-2 (IL-2), interleukin-6 (IL-6), interleukin-7 (IL-7), interleukin-17A (IL-17A), tumor necrosis factor-alpha (TNF-α), interferon-gamma (IFN-γ) (pg/mL), according to the manufacturer’s protocol. Plasma samples were prepared in 2-fold dilution using calibrator diluent. Using a flat-bottomed 96-well microplate, 25μl of premixed magnetic microparticles cocktail followed by 100μl of standards and diluted samples were added to each well. Analyte-specific antibodies were pre-coated onto colour-coded magnetic microparticles. Upon adding the standards and samples, immobilized capture antibodies coupled the analytes of interest. After 3 hours of incubation at room temperature on a horizontal orbital microplate shaker, the beads were washed three times using a magnetic device in order to wash away unbound substances. The premixed biotinylated antibody cocktail specific to the analytes of interest was then added to each well and the plate incubated for 1 hour at room temperature. The washing procedure was repeated to remove any unbound detection antibody. Next, fluorescently labelled streptavidin-phycoerythrin conjugate was added to each well and the plate incubated for 30 minutes followed by another washing step to remove any unbound streptavidin. Finally, the microparticles were re-suspended in wash buffer. After incubating for 2 minutes at room temperature, the microplate was read using the Luminex 200 instrument. All samples were assayed in duplicate and the results analyzed using SoftMax Pro 4.8 software which calculated the cytokines values following a 4-parameter fit.

*Bioinformatic Analyses*

For gene expression data, CEL files generated by the GeneAtlas scanner were imported into Partek Genomics Suite V6.6 for data visualization and quality control. All samples passed the quality criteria for hybridization controls, labelling controls and 3’/5’ Metrics. Background correction was conducted using Robust Multi-strip Average (RMA) ([Irizarry *et al*, 2003](#_ENREF_22)) to remove noise from auto fluorescence. Quantile Normalization ([Bolstad *et al*, 2003](#_ENREF_5)) was used to normalize the distribution of probe intensities among different microarray strips. Subsequently, a summarization step was conducted using a linear median polish algorithm ([Tukey, 1977](#_ENREF_47)) to integrate probe intensities and compute the expression levels for each gene transcript. Upon data upload, pre-processing of CEL data for the complete data set was performed using the Robust MultiChip Average ANOVA statistical test. To assess the effect of IFN-α treatment, a multiple linear contrast over time was performed, and gene lists obtained by applying cut-offs of both p-value (FDR corrected) of ≤0.05 and a minimum absolute fold change of 1.4. Ingenuity Pathway Analysis (IPA) Software was used to identify regulation of molecular signalling pathways. Baseline depression scores were used as a covariate in all bioinformatics analyses.

**S1** Real time PCR validation

|  | **Gene** | **FC Affymetrix** | **p value Affymetrix** | **FC**  **Real Time** | **p value Real Time** |
| --- | --- | --- | --- | --- | --- |
| **Genes differentially modulated at baseline between patients who develop and those who do not** | | | | | |
| solute carrier family 31 (copper transporters), member 1 | SLC31A1 | -1.4 | 1.55E-04 | -1.5 | p<0.001 |
| DnaJ (Hsp40) homolog. subfamily A. member 2 | DNAJA2 | 2.2 | 2.18E-06 | 2.3 | p<0.001 |
| metastasis associated lung adenocarcinoma transcript 1 | MALAT1 | 2.3 | 1.97E-05 | 2.6 | p<0.001 |
| **Genes modulated by 4 weeks of IFN-α in the whole sample** | | | | | |
| Interferon, alpha-inducible protein 27 | IFI27 | 32.1 | 7.68E-58 | 31.2 | p<0.001 |
| Interferon-induced protein 44-like | IFI44L | 11.6 | 3.92E-36 | 12.4 | p<0.001 |
| Ubiquitin specific peptidase 41 | USP41 | 8.4 | 2.21E-34 | 8.5 | p<0.001 |
| Radical S-adenosyl methionine domain containing 2 | RSAD2 | 7.2 | 1.07E-26 | 7.2 | p<0.001 |
| Interferon-induced protein 44 | IFI44 | 6.9 | 3.84E-33 | 7.1 | p<0.001 |
| Interferon-induced protein with tetratricopeptide repeats 1 | IFIT1 | 5.9 | 1.12E-24 | 6.5 | p<0.001 |

**S2** Genes differentially expressed at baseline in patients who develop depression compared to those who do not

| **Gene symbol** | **Gene assignment** | **p value** | **Fold change** |
| --- | --- | --- | --- |
| MALAT1 | metastasis associated lung adenocarcinoma transcript 1 (non-protein coding) | 1.97E-05 | 2.34 |
| DNAJA2 | DnaJ (Hsp40) homolog, subfamily A, member 2 | 2.18E-06 | 2.16 |
| TOB2 | transducer of ERBB2, 2 | 9.93E-05 | 2.00 |
| LLPH | LLP homolog, long-term synaptic facilitation (Aplysia) | 3.09E-04 | 1.90 |
| GNG2 | guanine nucleotide binding protein (G protein), gamma 2 | 4.19E-05 | 1.86 |
| SDAD1 | SDA1 domain containing 1 | 7.26E-05 | 1.68 |
| C3orf62 | chromosome 3 open reading frame 62 | 6.69E-08 | 1.62 |
| ROCK1 | Rho-associated, coiled-coil containing protein kinase 1 | 5.30E-04 | 1.60 |
| SNORA70G | small nucleolar RNA, H/ACA box 70G | 2.24E-04 | 1.60 |
| C10orf31 | chromosome 10 open reading frame 31 | 4.23E-04 | 1.59 |
| TMEM14E | transmembrane protein 14E | 9.40E-06 | 1.58 |
| SNORA41 | small nucleolar RNA, H/ACA box 41 | 1.40E-04 | 1.56 |
| RPL13A | ribosomal protein L13a | 4.49E-04 | 1.55 |
| IFNGR1 | interferon gamma receptor 1 | 2.01E-05 | 1.54 |
| NOL7 | nucleolar protein 7, 27kDa | 2.05E-04 | 1.53 |
| PDE4DIP | phosphodiesterase 4D interacting protein | 7.36E-05 | 1.48 |
| SNHG3 | small nucleolar RNA host gene 3 (non-protein coding) | 1.60E-04 | 1.48 |
| RNU6-79 | RNA, U6 small nuclear 79 | 8.61E-10 | 1.47 |
| RNF144B | ring finger protein 144B | 6.28E-06 | 1.46 |
| DLEU1 | deleted in lymphocytic leukemia 1 (non-protein coding) | 4.22E-06 | 1.43 |
| SNORD32B | small nucleolar RNA, C/D box 32B | 5.34E-04 | 1.41 |
| UFM1 | ubiquitin-fold modifier 1 | 2.47E-05 | 1.38 |
| RN5S343 | RNA, 5S ribosomal 343 | 8.38E-05 | 1.38 |
| SPDYE2 | speedy homolog E2 (Xenopus laevis) | 1.45E-04 | 1.37 |
| A2M | alpha-2-macroglobulin | 5.59E-05 | 1.36 |
| KRTAP10-9 | keratin associated protein 10-9 | 3.87E-05 | 1.35 |
| SNORA15 | small nucleolar RNA, H/ACA box 15 | 7.27E-07 | 1.35 |
| LINC00216 | long intergenic non-protein coding RNA 216 | 2.94E-05 | 1.33 |
| HCRP1 | hepatocellular carcinoma-related HCRP1 | 3.95E-05 | 1.33 |
| TSSK3 | testis-specific serine kinase 3 | 1.83E-04 | 1.32 |
| HIATL1 | hippocampus abundant transcript-like 1 | 2.24E-04 | 1.32 |
| DNAJC8 | DnaJ (Hsp40) homolog, subfamily C, member 8 | 1.74E-04 | 1.32 |
| FNDC9 | fibronectin type III domain containing 9 | 1.23E-04 | 1.31 |
| PZP | pregnancy-zone protein | 4.71E-05 | 1.30 |
| ZNF770 | zinc finger protein 770 | 2.98E-05 | 1.29 |
| HOXA2 | homeobox A2 | 7.75E-06 | 1.29 |
| PLA2G10 | phospholipase A2, group X | 2.22E-04 | 1.29 |
| C1orf192 | chromosome 1 open reading frame 192 | 6.36E-07 | 1.28 |
| LYSMD1 | LysM, putative peptidoglycan-binding, domain containing 1 | 1.60E-04 | 1.28 |
| ABHD12B | abhydrolase domain containing 12B | 1.64E-04 | 1.27 |
| GPX2 | glutathione peroxidase 2 (gastrointestinal) | 2.87E-04 | 1.27 |
| GNAT2 | guanine nucleotide binding protein (G protein), alpha transducing activity polypeptide 2 | 3.63E-05 | 1.26 |
| TMEM120B | transmembrane protein 120B | 2.59E-04 | 1.26 |
| GCNT7 | glucosaminyl (N-acetyl) transferase family member 7 | 3.97E-05 | 1.25 |
| OR10AD1 | olfactory receptor, family 10, subfamily AD, member 1 | 3.14E-08 | 1.24 |
| UGT2B10 | UDP glucuronosyltransferase 2 family, polypeptide B10 | 1.39E-05 | 1.23 |
| C1orf147 | chromosome 1 open reading frame 147 | 3.72E-04 | 1.23 |
| PPP3R1 | protein phosphatase 3, regulatory subunit B, alpha | 8.47E-05 | 1.22 |
| ZBED2 | zinc finger, BED-type containing 2 | 9.78E-05 | 1.22 |
| SERPIND1 | serpin peptidase inhibitor, clade D (heparin cofactor), member 1 | 3.75E-06 | 1.22 |
| LRRC18 | leucine rich repeat containing 18 | 4.63E-04 | 1.21 |
| SPDYE1 | speedy homolog E1 (Xenopus laevis) | 5.05E-04 | 1.21 |
| C17orf59 | chromosome 17 open reading frame 59 | 2.75E-04 | 1.21 |
| RN5S185 | RNA, 5S ribosomal 185 | 3.10E-04 | 1.20 |
| FAM115A | family with sequence similarity 115, member A | 4.09E-05 | 1.20 |
| TMEM185B | transmembrane protein 185B | 1.72E-04 | -1.21 |
| ZC3H6 | zinc finger CCCH-type containing 6 | 4.65E-04 | -1.22 |
| ZDHHC3 | zinc finger, DHHC-type containing 3 | 1.56E-04 | -1.23 |
| SKIL | SKI-like oncogene | 3.42E-04 | -1.23 |
| SENP1 | SUMO1/sentrin specific peptidase 1 | 7.77E-05 | -1.24 |
| PDCL | phosducin-like | 1.07E-04 | -1.24 |
| ZNF470 | zinc finger protein 470 | 1.37E-04 | -1.26 |
| SETD9 | SET domain containing 9 | 2.11E-05 | -1.26 |
| FGGY | FGGY carbohydrate kinase domain containing | 6.08E-05 | -1.27 |
| SNAP29 | synaptosomal-associated protein, 29kDa | 2.95E-04 | -1.27 |
| GSTM3 | glutathione S-transferase mu 3 (brain) | 5.27E-04 | -1.28 |
| PROS1 | protein S (alpha) | 9.52E-05 | -1.29 |
| LIMS1 | LIM and senescent cell antigen-like domains 1 | 1.47E-05 | -1.30 |
| NCK2 | NCK adaptor protein 2 | 3.23E-06 | -1.33 |
| SNX16 | sorting nexin 16 | 1.21E-04 | -1.35 |
| SLC31A1 | solute carrier family 31 (copper transporters), member 1 | 1.55E-04 | -1.37 |
| RGS18 | regulator of G-protein signaling 18 | 3.82E-04 | -1.38 |
| PAM | peptidylglycine alpha-amidating monooxygenase | 1.29E-05 | -1.42 |

**S3** Plasma cytokine results over time and in relation to depression development

|  | **Whole Sample** | **Time Effect** | **Depressed Patients** | **Non-Depressed Patients** | **Group Effect** | **Interaction Effect** |
| --- | --- | --- | --- | --- | --- | --- |
| IL-1β Baseline (pg/ml) | 2.41±0.11 |  | 2.44±0.14 | 2.40±0.15 |  |  |
| IL-1β TW4 (pg/ml) | 2.38±0.10 | *F*(1, 48) = 4.31, **p=0.043** | 2.41±0.13 | 2.35±0.13 | *F*(1, 48) = 0.01, p=0.9 | *F*(1, 48) = 0.79, p=0.4 |
| IL-1β TW24 (pg/ml) | 2.40±0.11 | *F*(1, 45) = 0.73, p=0.4 | 2.52±0.19 | 2.34±0.14 | *F*(1, 45) = 0.14, p=0.7 | *F*(1, 45) = 1.02, p=0.4 |
|  |  |  |  |  |  |  |
| IL-2 Baseline (pg/ml) | 3.53±0.07 |  | 3.63±0.13 | 3.48±0.09 |  |  |
| IL-2 TW4 (pg/ml) | 3.54±0.07 | *F*(1, 48) = 1.11, p=0.3 | 3.58±0.13 | 3.52±0.08 | *F*(1, 48) = 0.28, p=0.6 | *F*(1, 48) = 2.13, p=0.2 |
| IL-2 TW24 (pg/ml) | 3.55±0.07 | *F*(1, 45) = 0.66, p=0.4 | 3.59±0.13 | 3.53±0.09 | *F*(1, 45) = 0.02, p=0.9 | *F*(1, 45) = 0.26, p=0.6 |
|  |  |  |  |  |  |  |
| IL-6 Baseline (pg/ml) | 2.61±0.13 |  | 2.72±0.13 | 2.55±0.16 |  |  |
| IL-6 TW4 (pg/ml) | 2.89±0.15 | *F*(1, 47) = 6.75, **p=0.013** | 3.05±0.15 | 2.80±0.16 | *F*(1, 47) = 1.00, p=0.3 | *F*(1, 47) = 0.00, p=0.9 |
| IL-6 TW24 (pg/ml) | 3.01±0.13 | *F*(1, 44) = 6.90, **p=0.012** | 3.04±0.13 | 2.99±0.14 | *F*(1, 44) = 0.20, p=0.7 | *F*(1, 44) = 0.34, p=0.6 |
|  |  |  |  |  |  |  |
| IL-7 Baseline (pg/ml) | 1.96±0.13 |  | 2.02±0.24 | 1.93±0.14 |  |  |
| IL-7 TW4 (pg/ml) | 1.93±0.10 | *F*(1, 46) = 0.11, p=0.7 | 1.91±0.20 | 1.94±0.12 | *F*(1, 42) = 0.05, p=0.8 | *F*(1, 42) = 0.35, p=0.6 |
| IL-7 TW24 (pg/ml) | 1.87±0.12 | *F*(1, 45) = 0.73, p=0.4 | 1.70±0.17 | 1.95±0.15 | *F*(1, 42) = 0.74, p=0.4 | *F*(1, 42) = 0.00, p=0.9 |
|  |  |  |  |  |  |  |
| IL-17A Baseline (pg/ml) | 8.16±0.22 |  | 7.91±0.34 | 8.29±0.28 |  |  |
| IL-17A TW4 (pg/ml) | 8.30±0.20 | *F*(1, 47) = 5.60, **p=0.022** | 8.16±0.30 | 8.38±0.27 | *F*(1, 47) = 1.31, p=0.3 | *F*(1, 47) = 0.91, p=0.4 |
| IL-17A TW24 (pg/ml) | 8.37±0.21 | *F*(1, 44) = 7.81, **p=0.008** | 8.31±0.33 | 8.40±0.28 | *F*(1, 44) = 0.44, p=0.5 | *F*(1, 44) = 0.18, p=0.7 |
|  |  |  |  |  |  |  |
| TNF-α Baseline (pg/ml) | 5.88±0.36 |  | 5.72±0.61 | 5.98±0.46 |  |  |
| TNF-α TW4 (pg/ml) | 7.17±0.39 | *F*(1, 45) = 37.35, **p<0.001** | 7.58±0.69 | 6.95±0.48 | *F*(1, 45) = 0.03, p=0.9 | *F*(1, 45) = 1.11, p=0.3 |
| TNF-α TW24 (pg/ml) | 8.96±0.50 | *F*(1, 42) = 62.29, **p<0.001** | 9.99±0.82 | 8.43±0.61 | *F*(1, 42) = 0.23, p=0.6 | *F*(1, 42) = 3.67, p=0.1 |

**S4** Genes modulated by interferon-alpha at treatment week 4 specifically in patients who develop depression

| **Gene Symbol** | **Gene assignment** | **p value** | **Fold change** |
| --- | --- | --- | --- |
| HBZ | hemoglobin, zeta | 3.80E-03 | 3.25 |
| TSPAN7 | tetraspanin 7 | 3.75E-05 | 2.28 |
| CXCL10 | chemokine (C-X-C motif) ligand 10 | 4.24E-03 | 2.13 |
| HEMGN | hemogen | 4.95E-05 | 2.09 |
| USMG5 | up-regulated during skeletal muscle growth 5 homolog (mouse) | 4.09E-05 | 2.07 |
| RANBP10 | RAN binding protein 10 | 1.13E-05 | 2.00 |
| THEM5 | thioesterase superfamily member 5 | 4.25E-04 | 1.97 |
| CREG1 | cellular repressor of E1A-stimulated genes 1 | 2.32E-07 | 1.91 |
| IGF2BP2 | insulin-like growth factor 2 mRNA binding protein 2 | 7.86E-04 | 1.88 |
| STAP1 | signal transducing adaptor family member 1 | 3.30E-05 | 1.81 |
| BLOC1S1-RDH5 | BLOC1S1-RDH5 readthrough | 2.24E-06 | 1.80 |
| E2F2 | E2F transcription factor 2 | 2.80E-04 | 1.80 |
| TMOD1 | tropomodulin 1 | 2.63E-03 | 1.79 |
| MT1F | metallothionein 1F | 2.83E-09 | 1.79 |
| AHSP | alpha hemoglobin stabilizing protein | 6.12E-03 | 1.79 |
| TNFAIP6 | tumor necrosis factor, alpha-induced protein 6 | 1.52E-03 | 1.79 |
| BPGM | 2,3-bisphosphoglycerate mutase | 8.60E-04 | 1.78 |
| OSBP2 | oxysterol binding protein 2 | 8.32E-04 | 1.77 |
| GNA12 | guanine nucleotide binding protein (G protein) alpha 12 | 3.01E-05 | 1.76 |
| LSM3 | LSM3 homolog, U6 small nuclear RNA associated (S. cerevisiae) | 1.32E-03 | 1.76 |
| EVI2A | ecotropic viral integration site 2A | 1.06E-04 | 1.75 |
| KAT2B | K(lysine) acetyltransferase 2B | 3.63E-09 | 1.74 |
| TFDP1 | transcription factor Dp-1 | 6.36E-07 | 1.73 |
| CLIC2 | chloride intracellular channel 2 | 1.95E-04 | 1.73 |
| NFIA | nuclear factor I/A | 5.94E-06 | 1.72 |
| CD38 | CD38 molecule | 2.96E-06 | 1.72 |
| PSMA2 | proteasome (prosome, macropain) subunit, alpha type, 2 | 1.30E-04 | 1.72 |
| CD69 | CD69 molecule | 2.88E-07 | 1.71 |
| CD14 | CD14 molecule | 1.24E-07 | 1.71 |
| TMEM14B | transmembrane protein 14B | 6.72E-06 | 1.69 |
| AZI2 | 5-azacytidine induced 2 | 4.05E-12 | 1.69 |
| SLFN14 | schlafen family member 14 | 4.57E-03 | 1.69 |
| XPO7 | exportin 7 | 2.90E-06 | 1.68 |
| PPME1 | protein phosphatase methylesterase 1 | 1.42E-09 | 1.68 |
| RN5S403 | RN5S403 // RNA, 5S ribosomal 403 | 3.71E-06 | 1.67 |
| CRAT | carnitine O-acetyltransferase | 2.13E-04 | 1.67 |
| ALDH5A1 | aldehyde dehydrogenase 5 family, member A1 | 7.31E-04 | 1.66 |
| FECH | ferrochelatase | 6.55E-03 | 1.66 |
| DNAJA4 | DnaJ (Hsp40) homolog, subfamily A, member 4 | 4.91E-05 | 1.66 |
| FAM210B | family with sequence similarity 210, member B | 4.33E-04 | 1.66 |
| DNAJC15 | DnaJ (Hsp40) homolog, subfamily C, member 15 | 2.57E-06 | 1.66 |
| PLVAP | plasmalemma vesicle associated protein | 1.11E-03 | 1.65 |
| ANKH | ankylosis, progressive homolog (mouse) | 6.68E-06 | 1.65 |
| ARHGEF12 | Rho guanine nucleotide exchange factor (GEF) 12 | 1.90E-03 | 1.64 |
| RBBP8 | retinoblastoma binding protein 8 | 4.03E-09 | 1.63 |
| LRRCC1 | leucine rich repeat and coiled-coil centrosomal protein 1 | 1.03E-09 | 1.62 |
| ITLN1 | intelectin 1 (galactofuranose binding) | 1.09E-03 | 1.62 |
| C9orf78 | chromosome 9 open reading frame 78 | 2.88E-03 | 1.62 |
| RPL41 | ribosomal protein L41 | 4.69E-05 | 1.62 |
| ZC3H6 | zinc finger CCCH-type containing 6 | 7.66E-14 | 1.61 |
| IGFBP7 | insulin-like growth factor binding protein 7 | 2.21E-05 | 1.61 |
| TBCEL | tubulin folding cofactor E-like | 1.27E-04 | 1.61 |
| ATP5G1 | ATP synthase, H+ transporting, mitochondrial Fo complex, subunit C1 (subunit 9) | 7.39E-04 | 1.60 |
| TRAT1 | T cell receptor associated transmembrane adaptor 1 | 2.59E-04 | 1.60 |
| KLF1 | Kruppel-like factor 1 (erythroid) | 3.01E-03 | 1.60 |
| ZNF675 | zinc finger protein 675 | 3.42E-04 | 1.59 |
| TNFSF13B | tumor necrosis factor (ligand) superfamily, member 13b | 2.80E-06 | 1.59 |
| TAL1 | T-cell acute lymphocytic leukemia 1 | 1.38E-03 | 1.59 |
| C5orf4 | chromosome 5 open reading frame 4 | 6.43E-03 | 1.58 |
| SLFN5 | schlafen family member 5 | 6.34E-08 | 1.58 |
| PSMB6 | proteasome (prosome, macropain) subunit, beta type, 6 | 4.70E-04 | 1.58 |
| SLC7A5 | solute carrier family 7 (amino acid transporter light chain, L system), member 5 | 2.99E-03 | 1.57 |
| S100A8 | S100 calcium binding protein A8 | 4.15E-03 | 1.57 |
| IFI16 | interferon, gamma-inducible protein 16 | 6.38E-13 | 1.57 |
| LGALS2 | lectin, galactoside-binding, soluble, 2 | 6.52E-03 | 1.57 |
| SLC4A1 | solute carrier family 4, anion exchanger, member 1 | 5.17E-03 | 1.57 |
| SLC6A19 | solute carrier family 6 (neutral amino acid transporter), member 19 | 3.90E-03 | 1.57 |
| TMCC2 | transmembrane and coiled-coil domain family 2 | 2.29E-05 | 1.56 |
| COX16 | COX16 cytochrome c oxidase assembly homolog (S. cerevisiae) | 1.14E-03 | 1.56 |
| USP12 | ubiquitin specific peptidase 12 | 2.25E-04 | 1.55 |
| PML | promyelocytic leukemia | 4.97E-07 | 1.55 |
| SNX2 | sorting nexin 2 | 6.78E-07 | 1.55 |
| TPGS2 | tubulin polyglutamylase complex subunit 2 | 7.09E-05 | 1.54 |
| RFESD | Rieske (Fe-S) domain containing | 1.13E-05 | 1.54 |
| WDR67 | WD repeat domain 67 | 4.23E-10 | 1.54 |
| SLIRP | SRA stem-loop interacting RNA binding protein | 3.74E-04 | 1.54 |
| MARCH2 | membrane-associated ring finger (C3HC4) 2, E3 ubiquitin protein ligase | 8.84E-04 | 1.54 |
| DNAJC6 | DnaJ (Hsp40) homolog, subfamily C, member 6 | 2.06E-04 | 1.54 |
| ZNF260 | zinc finger protein 260 | 5.96E-05 | 1.53 |
| SPECC1 | sperm antigen with calponin homology and coiled-coil domains 1 | 6.59E-06 | 1.53 |
| FAM190A | family with sequence similarity 190, member A | 7.00E-05 | 1.53 |
| ANP32B | acidic (leucine-rich) nuclear phosphoprotein 32 family, member B | 1.42E-06 | 1.53 |
| GABPB2 | GA binding protein transcription factor, beta subunit 2 | 1.59E-10 | 1.52 |
| TMSB10 | thymosin beta 10 | 1.92E-05 | 1.52 |
| HCST | hematopoietic cell signal transducer | 1.63E-04 | 1.51 |
| AIF1 | allograft inflammatory factor 1 | 2.37E-03 | 1.51 |
| ABCG2 | ATP-binding cassette, sub-family G (WHITE), member 2 | 4.09E-04 | 1.51 |
| SSBP3 | single stranded DNA binding protein 3 | 5.76E-05 | 1.51 |
| CD48 | CD48 molecule | 2.02E-05 | 1.51 |
| HSPB1 | heat shock 27kDa protein 1 | 2.27E-04 | 1.51 |
| MCM8 | minichromosome maintenance complex component 8 | 5.82E-06 | 1.51 |
| SP110 | SP110 nuclear body protein | 1.99E-10 | 1.51 |
| PEAK1 | NKF3 kinase family member | 3.14E-09 | 1.51 |
| TRAK2 | trafficking protein, kinesin binding 2 | 2.95E-04 | 1.50 |
| BCL2L1 | BCL2-like 1 | 3.92E-04 | 1.50 |
| MARCH3 | membrane-associated ring finger (C3HC4) 3, E3 ubiquitin protein ligase | 7.68E-04 | 1.50 |
| HINT2 | histidine triad nucleotide binding protein 2 | 9.21E-05 | 1.50 |
| STOM | stomatin | 1.00E-06 | 1.49 |
| NRIP1 | nuclear receptor interacting protein 1 | 1.89E-06 | 1.49 |
| ATP10A | ATPase, class V, type 10A | 7.99E-12 | 1.49 |
| FMNL2 | formin-like 2 | 6.75E-06 | 1.49 |
| MZT2B | mitotic spindle organizing protein 2B | 3.95E-06 | 1.48 |
| COMMD6 | COMM domain containing 6 | 5.63E-03 | 1.48 |
| RPL5 | ribosomal protein L5 | 4.45E-03 | 1.48 |
| SCARB2 | scavenger receptor class B, member 2 | 2.62E-07 | 1.48 |
| ERMAP | erythroblast membrane-associated protein (Scianna blood group) | 3.17E-04 | 1.48 |
| NUP62CL | nucleoporin 62kDa C-terminal like | 6.35E-07 | 1.48 |
| IFI27L2 | interferon, alpha-inducible protein 27-like 2 | 6.52E-08 | 1.48 |
| GPD2 | glycerol-3-phosphate dehydrogenase 2 (mitochondrial) | 9.10E-06 | 1.48 |
| CLTB | clathrin, light chain B | 6.98E-04 | 1.48 |
| CMAS | cytidine monophosphate N-acetylneuraminic acid synthetase | 9.49E-06 | 1.48 |
| ARV1 | ARV1 homolog (S. cerevisiae) | 7.34E-06 | 1.48 |
| TFEC | transcription factor EC | 3.51E-04 | 1.47 |
| MAFB | v-maf musculoaponeurotic fibrosarcoma oncogene homolog B (avian) | 3.06E-06 | 1.47 |
| ZNF79 | zinc finger protein 79 | 1.79E-12 | 1.47 |
| PLSCR4 | phospholipid scramblase 4 | 1.02E-07 | 1.47 |
| SEC62 | SEC62 homolog (S. cerevisiae) | 5.70E-06 | 1.47 |
| ZNF480 | zinc finger protein 480 | 1.72E-04 | 1.47 |
| UBQLNL | ubiquilin-like | 5.11E-10 | 1.47 |
| LGALS9C | lectin, galactoside-binding, soluble, 9C | 2.94E-06 | 1.46 |
| DCP1B | DCP1 decapping enzyme homolog B (S. cerevisiae) | 2.89E-09 | 1.46 |
| ATP5E | ATP synthase, H+ transporting, mitochondrial F1 complex, epsilon subunit | 8.04E-05 | 1.46 |
| THOC7 | THO complex 7 homolog (Drosophila) | 5.96E-06 | 1.46 |
| ZNF496 | zinc finger protein 496 | 2.62E-09 | 1.46 |
| ATXN7L1 | ataxin 7-like 1 | 3.71E-07 | 1.46 |
| GTPBP2 | GTP binding protein 2 | 2.52E-08 | 1.46 |
| RAB6A | RAB6A, member RAS oncogene family | 1.55E-05 | 1.46 |
| TIMMDC1 | translocase of inner mitochondrial membrane domain containing 1 | 3.24E-06 | 1.46 |
| NME4 | NME/NM23 nucleoside diphosphate kinase 4 | 2.00E-05 | 1.46 |
| BMP2K | BMP2 inducible kinase | 3.93E-06 | 1.45 |
| DDI2 | DNA-damage inducible 1 homolog 2 (S. cerevisiae) | 1.81E-08 | 1.45 |
| ECH1 | enoyl CoA hydratase 1, peroxisomal | 3.47E-06 | 1.45 |
| TUBG1 | tubulin, gamma 1 | 7.28E-09 | 1.45 |
| MXI1 | MAX interactor 1 | 3.14E-03 | 1.45 |
| ADAL | adenosine deaminase-like | 1.45E-05 | 1.45 |
| LIN9 | lin-9 homolog (C. elegans) | 2.68E-05 | 1.45 |
| MAP4K5 | mitogen-activated protein kinase kinase kinase kinase 5 | 1.28E-07 | 1.45 |
| PMAIP1 | phorbol-12-myristate-13-acetate-induced protein 1 | 1.19E-05 | 1.45 |
| ZNF382 | zinc finger protein 382 | 1.68E-03 | 1.45 |
| PTP4A1 | protein tyrosine phosphatase type IVA, member 1 | 3.09E-08 | 1.45 |
| TRIM38 | tripartite motif containing 38 | 5.79E-09 | 1.45 |
| WNK1 | WNK lysine deficient protein kinase 1 | 1.18E-04 | 1.44 |
| BMPR2 | bone morphogenetic protein receptor, type II (serine/threonine kinase) | 9.39E-07 | 1.44 |
| SLC18B1 | solute carrier family 18, subfamily B, member 1 | 1.70E-06 | 1.44 |
| MOP-1 | MOP-1 | 2.09E-03 | 1.44 |
| ABCC4 | ATP-binding cassette, sub-family C (CFTR/MRP), member 4 | 7.33E-04 | 1.44 |
| GBP3 | guanylate binding protein 3 | 7.67E-05 | 1.44 |
| RPS15 | ribosomal protein S15 | 1.25E-05 | 1.44 |
| MGST1 | microsomal glutathione S-transferase 1 | 8.02E-05 | 1.44 |
| RBM11 | RNA binding motif protein 11 | 2.72E-06 | 1.44 |
| ZNF441 | zinc finger protein 441 | 1.18E-04 | 1.44 |
| CCRL2 | chemokine (C-C motif) receptor-like 2 | 3.58E-08 | 1.44 |
| ORC3 | origin recognition complex, subunit 3 | 5.95E-07 | 1.44 |
| PCGF5 | polycomb group ring finger 5 | 1.70E-15 | 1.44 |
| ELL2 | elongation factor, RNA polymerase II, 2 | 1.38E-06 | 1.44 |
| KIAA1586 | KIAA1586 | 8.41E-04 | 1.44 |
| ZNF737 | zinc finger protein 737 | 5.79E-04 | 1.44 |
| AKT2 | v-akt murine thymoma viral oncogene homolog 2 | 2.74E-06 | 1.44 |
| ACP1 | acid phosphatase 1, soluble | 4.18E-04 | 1.44 |
| SP140 | SP140 nuclear body protein | 2.58E-06 | 1.44 |
| GLRX5 | glutaredoxin 5 | 8.00E-03 | 1.43 |
| ZNF83 | zinc finger protein 83 | 2.06E-03 | 1.43 |
| FURIN | furin (paired basic amino acid cleaving enzyme) | 6.98E-04 | 1.43 |
| SUB1 | SUB1 homolog (S. cerevisiae) | 5.99E-05 | 1.43 |
| RASGEF1B | RasGEF domain family, member 1B | 1.68E-05 | 1.43 |
| ZMAT1 | zinc finger, matrin-type 1 | 3.45E-04 | 1.43 |
| AKAP7 | A kinase (PRKA) anchor protein 7 | 4.14E-08 | 1.43 |
| ZFAND4 | zinc finger, AN1-type domain 4 | 1.09E-05 | 1.43 |
| ALG13 | asparagine-linked glycosylation 13 homolog (S. cerevisiae) | 2.21E-05 | 1.43 |
| SLC44A4 | solute carrier family 44, member 4 | 1.43E-03 | 1.43 |
| CDK8 | cyclin-dependent kinase 8 | 1.32E-06 | 1.43 |
| MINPP1 | multiple inositol-polyphosphate phosphatase 1 | 8.72E-06 | 1.43 |
| TMA7 | translational machinery associated 7 homolog (S. cerevisiae) | 5.69E-05 | 1.43 |
| ERH | enhancer of rudimentary homolog (Drosophila) | 7.91E-04 | 1.43 |
| RPH3A | rabphilin 3A homolog (mouse) | 1.01E-05 | 1.43 |
| GALM | galactose mutarotase (aldose 1-epimerase) | 3.49E-04 | 1.43 |
| RANBP9 | RAN binding protein 9 | 1.03E-08 | 1.43 |
| C8orf31 | chromosome 8 open reading frame 31 | 3.63E-04 | 1.42 |
| DENND4A | DENN/MADD domain containing 4A | 1.50E-07 | 1.42 |
| GAB1 | GRB2-associated binding protein 1 | 5.36E-04 | 1.42 |
| MT1X | metallothionein 1X | 4.15E-09 | 1.42 |
| WTH3DI | RAB6C-like | 4.47E-05 | 1.42 |
| RNF213 | ring finger protein 213 | 9.55E-06 | 1.42 |
| MARCH8 | membrane-associated ring finger (C3HC4) 8, E3 ubiquitin protein ligase | 1.23E-03 | 1.42 |
| MGST3 | microsomal glutathione S-transferase 3 | 3.78E-03 | 1.42 |
| EPB41L4A | erythrocyte membrane protein band 4.1 like 4A | 6.22E-05 | 1.42 |
| FARSB | phenylalanyl-tRNA synthetase, beta subunit | 5.60E-08 | 1.42 |
| RASGRP3 | RAS guanyl releasing protein 3 (calcium and DAG-regulated) | 1.89E-04 | 1.42 |
| FAM46A | family with sequence similarity 46, member A | 1.03E-05 | 1.42 |
| ACOT13 | acyl-CoA thioesterase 13 | 3.47E-08 | 1.42 |
| NOB1 | NIN1/RPN12 binding protein 1 homolog (S. cerevisiae) | 5.93E-06 | 1.42 |
| GIMAP1 | GTPase, IMAP family member 1 | 2.82E-06 | 1.42 |
| STX17 | syntaxin 17 | 1.04E-09 | 1.42 |
| LPAR6 | lysophosphatidic acid receptor 6 | 6.85E-03 | 1.42 |
| UBAP2 | ubiquitin associated protein 2 | 1.40E-09 | 1.42 |
| COPS2 | COP9 constitutive photomorphogenic homolog subunit 2 (Arabidopsis) | 3.07E-04 | 1.42 |
| METTL7A | methyltransferase like 7A | 5.13E-04 | 1.41 |
| TTC21A | tetratricopeptide repeat domain 21A | 1.42E-06 | 1.41 |
| ANKRD45 | ankyrin repeat domain 45 | 2.40E-06 | 1.41 |
| BOLA3 | bolA homolog 3 (E. coli) | 7.53E-07 | 1.41 |
| IFI30 | interferon, gamma-inducible protein 30 | 1.68E-05 | 1.41 |
| RNF187 | ring finger protein 187 | 5.34E-04 | 1.41 |
| MSMO1 | methylsterol monooxygenase 1 | 4.22E-05 | 1.41 |
| KNTC1 | kinetochore associated 1 | 1.53E-08 | 1.41 |
| AK5 | adenylate kinase 5 | 2.04E-03 | 1.41 |
| MRPS24 | mitochondrial ribosomal protein S24 | 6.97E-05 | 1.41 |
| PDHX | pyruvate dehydrogenase complex, component X | 1.05E-07 | 1.41 |
| POMP | proteasome maturation protein | 4.01E-05 | 1.41 |
| ODF2L | outer dense fiber of sperm tails 2-like | 4.01E-04 | 1.41 |
| GCLC | glutamate-cysteine ligase, catalytic subunit | 1.13E-04 | 1.41 |
| IL1RN | interleukin 1 receptor antagonist | 1.04E-03 | 1.41 |
| IL6ST | interleukin 6 signal transducer (gp130, oncostatin M receptor) | 4.70E-06 | 1.41 |
| ZMAT2 | zinc finger, matrin-type 2 | 1.10E-03 | 1.41 |
| C1orf31 | chromosome 1 open reading frame 31 | 6.83E-05 | 1.41 |
| ANKRD36B | ankyrin repeat domain 36B | 2.03E-03 | 1.41 |
| FAM100A | family with sequence similarity 100, member A | 3.79E-03 | 1.40 |
| LGALS3 | lectin, galactoside-binding, soluble, 3 | 5.19E-04 | 1.40 |
| RNF123 | ring finger protein 123 | 8.90E-03 | 1.40 |
| SHISA7 | shisa homolog 7 (Xenopus laevis) | 5.36E-04 | 1.40 |
| ZNF92 | zinc finger protein 92 | 1.68E-05 | 1.40 |
| HESX1 | HESX homeobox 1 | 1.43E-08 | 1.40 |
| CDC27 | cell division cycle 27 homolog (S. cerevisiae) | 2.81E-09 | 1.40 |
| ZNF493 | zinc finger protein 493 | 9.89E-04 | 1.40 |
| SLC2A1 | solute carrier family 2 (facilitated glucose transporter), member 1 | 8.43E-04 | 1.40 |
| TMX4 | thioredoxin-related transmembrane protein 4 | 1.12E-09 | -1.40 |
| ZNFX1-AS1 | ZNFX1 antisense RNA 1 (non-protein coding) | 7.94E-10 | -1.40 |
| NCF1 | neutrophil cytosolic factor 1 | 3.52E-06 | -1.40 |
| PDK3 | pyruvate dehydrogenase kinase, isozyme 3 | 1.77E-08 | -1.40 |
| C10orf54 | chromosome 10 open reading frame 54 | 1.46E-07 | -1.40 |
| TNFRSF14 | tumor necrosis factor receptor superfamily, member 14 | 2.41E-11 | -1.40 |
| PZP | pregnancy-zone protein | 5.37E-07 | -1.40 |
| SNX10 | sorting nexin 10 | 1.16E-05 | -1.40 |
| PCMTD2 | protein-L-isoaspartate (D-aspartate) O-methyltransferase domain containing 2 | 1.70E-06 | -1.40 |
| TLR6 | toll-like receptor 6 | 1.33E-04 | -1.41 |
| F2RL1 | coagulation factor II (thrombin) receptor-like 1 | 1.14E-04 | -1.41 |
| PPP3CA | protein phosphatase 3, catalytic subunit, alpha isozyme | 2.06E-11 | -1.41 |
| ATG16L2 | autophagy related 16-like 2 (S. cerevisiae) | 1.45E-06 | -1.41 |
| CAPNS2 | calpain, small subunit 2 | 9.10E-05 | -1.41 |
| ZNF238 | zinc finger protein 238 | 4.82E-10 | -1.41 |
| FAM126B | family with sequence similarity 126, member B | 5.75E-05 | -1.41 |
| ELOVL5 | ELOVL fatty acid elongase 5 | 7.85E-13 | -1.41 |
| KLHL2 | kelch-like 2, Mayven (Drosophila) | 1.57E-05 | -1.41 |
| TBC1D2B | TBC1 domain family, member 2B | 5.57E-07 | -1.41 |
| HK2 | hexokinase 2 | 2.71E-05 | -1.41 |
| RGL2 | ral guanine nucleotide dissociation stimulator-like 2 | 1.14E-09 | -1.41 |
| SLC16A3 | solute carrier family 16, member 3 (monocarboxylic acid transporter), member 3 | 2.20E-06 | -1.41 |
| RFWD2 | ring finger and WD repeat domain 2, E3 ubiquitin protein ligase | 7.97E-08 | -1.41 |
| CHD9 | Achromodomain helicase DNA binding protein 9 | 4.09E-10 | -1.41 |
| CXCR1 | chemokine (C-X-C motif) receptor 1 | 5.17E-05 | -1.41 |
| TRIM13 | tripartite motif containing 13 | 2.31E-07 | -1.41 |
| NRG1 | neuregulin 1 | 3.96E-04 | -1.41 |
| SPDYE1 | speedy homolog E1 (Xenopus laevis) | 6.60E-08 | -1.42 |
| BTNL8 | butyrophilin-like 8 | 1.08E-05 | -1.42 |
| RAB11FIP4 | RAB11 family interacting protein 4 (class II) | 6.83E-10 | -1.42 |
| MAEA | macrophage erythroblast attacher | 5.84E-11 | -1.42 |
| IKBIP | IKBKB interacting protein | 1.21E-04 | -1.42 |
| SIRPA | signal-regulatory protein alpha | 2.93E-09 | -1.42 |
| SNAP23 | synaptosomal-associated protein, 23kDa | 2.56E-08 | -1.42 |
| FOS | FBJ murine osteosarcoma viral oncogene homolog | 4.50E-04 | -1.42 |
| C1orf192 | chromosome 1 open reading frame 192 | 7.23E-11 | -1.42 |
| EPHB4 | EPH receptor B4 | 3.38E-07 | -1.42 |
| LPGAT1 | lysophosphatidylglycerol acyltransferase 1 | 1.76E-08 | -1.42 |
| RNF24 | ring finger protein 24 | 2.31E-05 | -1.42 |
| GNAT2 | guanine nucleotide binding protein (G protein), alpha transducing sctivity polypeptide 2 | 1.04E-08 | -1.42 |
| ZNF552 | zinc finger protein 552 | 6.50E-08 | -1.42 |
| TAF1D | TATA box binding protein (TBP)-associated factor, RNA polymerase I, D, 41kDa | 1.40E-03 | -1.42 |
| RNF149 | ring finger protein 149 | 6.54E-06 | -1.42 |
| PADI4 | peptidyl arginine deiminase, type IV | 1.10E-03 | -1.42 |
| PISD | phosphatidylserine decarboxylase | 3.23E-08 | -1.42 |
| PLEKHG3 | pleckstrin homology domain containing, family G (with RhoGef domain), member 3 | 3.70E-07 | -1.42 |
| TBXAS1 | thromboxane A synthase 1 (platelet) | 6.74E-09 | -1.42 |
| SCAP | SREBF chaperone | 4.51E-15 | -1.42 |
| SPAG9 | sperm associated antigen 9 | 1.21E-09 | -1.42 |
| PGD | phosphogluconate dehydrogenase | 1.26E-05 | -1.42 |
| SPATA6 | spermatogenesis associated 6 | 5.65E-07 | -1.43 |
| TMEM43 | transmembrane protein 43 | 2.61E-10 | -1.43 |
| IL1B | interleukin 1, beta | 7.37E-04 | -1.43 |
| IER3 | immediate early response 3 | 9.61E-06 | -1.43 |
| SCARNA6 | small Cajal body-specific RNA 6 | 9.21E-03 | -1.43 |
| FAM63A | family with sequence similarity 63, member A | 4.08E-07 | -1.43 |
| PSTPIP2 | proline-serine-threonine phosphatase interacting protein 2 | 6.50E-05 | -1.43 |
| WDFY3 | WD repeat and FYVE domain containing 3 | 4.95E-04 | -1.43 |
| RNASET2 | ribonuclease T2 | 1.02E-06 | -1.43 |
| NRBF2 | nuclear receptor binding factor 2 | 5.99E-06 | -1.43 |
| SSH2 | slingshot homolog 2 (Drosophila) | 5.85E-08 | -1.43 |
| ABHD12B | abhydrolase domain containing 12B | 1.33E-07 | -1.43 |
| ALPK1 | alpha-kinase 1 | 2.47E-04 | -1.43 |
| PTGS2 | prostaglandin-endoperoxide synthase 2 (prostaglandin G/H synthase and cyclooxygenase) | 7.62E-04 | -1.43 |
| ADAM19 | ADAM metallopeptidase domain 19 | 9.79E-08 | -1.43 |
| DPEP2 | dipeptidase 2 | 2.72E-08 | -1.43 |
| SNHG10 | small nucleolar RNA host gene 10 (non-protein coding) | 9.23E-03 | -1.43 |
| SORD | sorbitol dehydrogenase | 3.10E-07 | -1.43 |
| HAUS4 | HAUS augmin-like complex, subunit 4 | 1.09E-04 | -1.43 |
| CYB5R4 | cytochrome b5 reductase 4 | 1.06E-07 | -1.43 |
| EEF2 | eukaryotic translation elongation factor 2 | 2.09E-14 | -1.43 |
| KIAA0232 | KIAA0232 | 2.38E-10 | -1.44 |
| SNORD105 | small nucleolar RNA, C/D box 105 | 1.86E-03 | -1.44 |
| LSP1 | lymphocyte-specific protein 1 | 2.87E-08 | -1.44 |
| CD46 | CD46 molecule, complement regulatory protein | 7.11E-10 | -1.44 |
| RRM2B | ribonucleotide reductase M2 B (TP53 inducible) | 6.12E-07 | -1.44 |
| KRT23 | keratin 23 (histone deacetylase inducible) | 5.89E-03 | -1.44 |
| MYH9 | myosin, heavy chain 9, non-muscle | 3.02E-05 | -1.44 |
| PELI1 | pellino E3 ubiquitin protein ligase 1 | 1.05E-04 | -1.44 |
| C7orf49 | chromosome 7 open reading frame 49 | 7.89E-08 | -1.44 |
| HCG27 | HLA complex group 27 (non-protein coding) | 4.02E-07 | -1.44 |
| ZNF467 | zinc finger protein 467 | 2.72E-06 | -1.44 |
| PLCG2 | phospholipase C, gamma 2 (phosphatidylinositol-specific) | 6.58E-08 | -1.44 |
| DENND3 | DENN/MADD domain containing 3 | 2.61E-06 | -1.44 |
| TECPR2 | tectonin beta-propeller repeat containing 2 | 1.00E-04 | -1.45 |
| EPS15L1 | epidermal growth factor receptor pathway substrate 15-like 1 | 2.50E-11 | -1.45 |
| CDC123 | cell division cycle 123 homolog (S. cerevisiae) | 1.47E-14 | -1.45 |
| KIF13A | kinesin family member 13A | 6.01E-05 | -1.45 |
| AQP9 | aquaporin 9 | 1.01E-05 | -1.45 |
| IL6R | interleukin 6 receptor | 3.05E-09 | -1.45 |
| MAP3K3 | mitogen-activated protein kinase kinase kinase 3 | 1.57E-07 | -1.45 |
| DUSP1 | dual specificity phosphatase 1 | 2.27E-06 | -1.45 |
| CPQ | carboxypeptidase Q | 3.66E-08 | -1.45 |
| DEF8 | differentially expressed in FDCP 8 homolog (mouse) | 4.15E-13 | -1.45 |
| FLOT1 | flotillin 1 | 1.43E-06 | -1.45 |
| RAB5B | RAB5B, member RAS oncogene family | 6.85E-15 | -1.45 |
| CMTM2 | CKLF-like MARVEL transmembrane domain containing 2 | 1.75E-07 | -1.45 |
| CYBRD1 | cytochrome b reductase 1 | 4.49E-03 | -1.45 |
| MSRB1 | methionine sulfoxide reductase B1 | 9.20E-07 | -1.45 |
| C5AR1 | complement component 5a receptor 1 | 5.62E-05 | -1.46 |
| RPA2 | replication protein A2, 32kDa | 9.41E-10 | -1.46 |
| SLC46A2 | solute carrier family 46, member 2 | 7.37E-09 | -1.46 |
| SIPA1L1 | signal-induced proliferation-associated 1 like 1 | 2.04E-07 | -1.46 |
| PFKFB4 | 6-phosphofructo-2-kinase/fructose-2,6-biphosphatase 4 | 8.18E-06 | -1.46 |
| MBOAT1 | membrane bound O-acyltransferase domain containing 1 | 3.86E-07 | -1.46 |
| AHCTF1 | AT hook containing transcription factor 1 | 1.27E-07 | -1.46 |
| LRRK2 | leucine-rich repeat kinase 2 | 1.41E-04 | -1.46 |
| FPR2 | formyl peptide receptor 2 | 7.92E-04 | -1.46 |
| PLXNC1 | plexin C1 | 1.82E-06 | -1.46 |
| OTUD1 | OTU domain containing 1 | 3.88E-09 | -1.46 |
| EYS | eyes shut homolog (Drosophila) | 5.34E-05 | -1.46 |
| CDC42EP2 | CDC42 effector protein (Rho GTPase binding) 2 | 2.62E-06 | -1.46 |
| KREMEN1 | kringle containing transmembrane protein 1 | 1.85E-03 | -1.47 |
| DNAJC8 | DnaJ (Hsp40) homolog, subfamily C, member 8 | 8.87E-07 | -1.47 |
| SLC19A1 | solute carrier family 19 (folate transporter), member 1 | 1.01E-08 | -1.47 |
| PHC2 | polyhomeotic homolog 2 (Drosophila) | 2.69E-08 | -1.47 |
| EIF4A1 | eukaryotic translation initiation factor 4A1 | 7.48E-05 | -1.47 |
| MYO1F | myosin IF | 4.67E-08 | -1.47 |
| LINC00216 | long intergenic non-protein coding RNA 216 | 1.26E-07 | -1.47 |
| SYK | spleen tyrosine kinase | 4.80E-10 | -1.47 |
| A2M | alpha-2-macroglobulin | 2.00E-06 | -1.47 |
| MBD6 | methyl-CpG binding domain protein 6 | 6.25E-12 | -1.47 |
| SLC15A3 | solute carrier family 15, member 3 | 6.92E-09 | -1.48 |
| HCRP1 | hepatocellular carcinoma-related HCRP1 | 1.10E-07 | -1.48 |
| KIAA0513 | KIAA0513 | 2.18E-08 | -1.48 |
| TMEM88 | transmembrane protein 88 | 1.15E-08 | -1.48 |
| CSF2RA | colony stimulating factor 2 receptor, alpha, low-affinity (granulocyte-macrophage) | 3.50E-05 | -1.48 |
| HRH2 | histamine receptor H2 | 4.53E-06 | -1.48 |
| PRKAR1A | protein kinase, cAMP-dependent, regulatory, type I, alpha | 1.66E-08 | -1.48 |
| C14orf43 | chromosome 14 open reading frame 43 | 1.96E-08 | -1.48 |
| NPEPPS | aminopeptidase puromycin sensitive | 1.09E-10 | -1.48 |
| IDS | iduronate 2-sulfatase | 1.80E-12 | -1.48 |
| PPP4R1 | protein phosphatase 4, regulatory subunit 1 | 1.28E-06 | -1.49 |
| NCF4 | neutrophil cytosolic factor 4, 40kDa | 9.40E-07 | -1.49 |
| INPP5A | inositol polyphosphate-5-phosphatase, 40kDa | 2.54E-09 | -1.49 |
| NLRP12 | NLR family, pyrin domain containing 12 | 2.34E-06 | -1.49 |
| RNU6-79 | RNA, U6 small nuclear 79 | 1.40E-09 | -1.49 |
| HEATR5A | HEAT repeat containing 5A | 2.81E-10 | -1.49 |
| GCA | grancalcin, EF-hand calcium binding protein | 2.25E-05 | -1.49 |
| TM6SF1 | transmembrane 6 superfamily member 1 | 1.16E-06 | -1.49 |
| MAST3 | microtubule associated serine/threonine kinase 3 | 1.76E-12 | -1.49 |
| NQO2 | NAD(P)H dehydrogenase, quinone 2 | 1.93E-05 | -1.49 |
| OXER1 | oxoeicosanoid (OXE) receptor 1 | 1.42E-07 | -1.49 |
| SNORA14B | small nucleolar RNA, H/ACA box 14B | 7.69E-03 | -1.49 |
| HIATL1 | hippocampus abundant transcript-like 1 | 5.57E-07 | -1.50 |
| ALDH1A1 | aldehyde dehydrogenase 1 family, member A1 | 1.66E-04 | -1.50 |
| ZBTB34 | zinc finger and BTB domain containing 34 | 9.70E-09 | -1.50 |
| ERGIC1 | endoplasmic reticulum-golgi intermediate compartment (ERGIC) 1 | 8.02E-11 | -1.50 |
| CXCR2 | chemokine (C-X-C motif) receptor 2 | 4.64E-05 | -1.50 |
| SGK1 | serum/glucocorticoid regulated kinase 1 | 1.84E-06 | -1.50 |
| ALDH2 | aldehyde dehydrogenase 2 family (mitochondrial) | 2.08E-04 | -1.50 |
| RELL1 | RELT-like 1 | 3.72E-13 | -1.51 |
| SNORA38 | small nucleolar RNA, H/ACA box 38 | 5.56E-06 | -1.51 |
| CEP19 | centrosomal protein 19kDa | 3.02E-04 | -1.51 |
| ZNF185 | zinc finger protein 185 (LIM domain) | 1.17E-03 | -1.51 |
| RALB | v-ral simian leukemia viral oncogene homolog B | 5.12E-05 | -1.51 |
| PPP1R8 | protein phosphatase 1, regulatory subunit 8 | 9.75E-07 | -1.51 |
| ZNF587 | zinc finger protein 587 | 3.19E-07 | -1.51 |
| AGPAT9 | 1-acylglycerol-3-phosphate O-acyltransferase 9 | 3.95E-05 | -1.51 |
| REPS2 | RALBP1 associated Eps domain containing 2 | 7.48E-06 | -1.52 |
| TSSK3 | testis-specific serine kinase 3 | 2.13E-07 | -1.52 |
| AOC3 | amine oxidase, copper containing 3 (vascular adhesion protein 1) | 7.12E-07 | -1.52 |
| GAFA2 | FGF-2 activity-associated protein 2 | 1.80E-05 | -1.52 |
| SLC43A2 | solute carrier family 43, member 2 | 2.02E-09 | -1.52 |
| PGS1 | phosphatidylglycerophosphate synthase 1 | 3.81E-09 | -1.52 |
| MPZL3 | myelin protein zero-like 3 | 3.34E-08 | -1.52 |
| LINC00266-1 | long intergenic non-protein coding RNA 266-1 | 1.45E-07 | -1.52 |
| RLIM | ring finger protein, LIM domain interacting | 1.73E-06 | -1.52 |
| XKR8 | XK, Kell blood group complex subunit-related family, member 8 | 6.30E-16 | -1.52 |
| ATP6V1A | ATPase, H+ transporting, lysosomal 70kDa, V1 subunit A | 6.72E-06 | -1.52 |
| RPPH1 | ribonuclease P RNA component H1 | 4.21E-03 | -1.52 |
| TBL1X | transducin (beta)-like 1X-linked | 1.19E-08 | -1.53 |
| LYSMD1 | LysM, putative peptidoglycan-binding, domain containing 1 | 2.64E-09 | -1.53 |
| SNORA74A | small nucleolar RNA, H/ACA box 74A | 4.46E-05 | -1.53 |
| LPPR2 | lipid phosphate phosphatase-related protein type 2 | 1.76E-07 | -1.53 |
| PID1 | phosphotyrosine interaction domain containing 1 | 8.87E-11 | -1.53 |
| SIRPD | signal-regulatory protein delta | 2.46E-08 | -1.53 |
| CYSLTR2 | cysteinyl leukotriene receptor 2 | 1.22E-03 | -1.53 |
| ABHD5 | abhydrolase domain containing 5 | 3.32E-07 | -1.53 |
| ARHGAP9 | Rho GTPase activating protein 9 | 1.62E-11 | -1.53 |
| DDX11L2 | DEAD/H (Asp-Glu-Ala-Asp/His) box helicase 11 like 2 | 1.72E-03 | -1.54 |
| EEPD1 | endonuclease/exonuclease/phosphatase family domain containing 1 | 2.72E-08 | -1.54 |
| SNORA15 | small nucleolar RNA, H/ACA box 15 | 4.20E-10 | -1.54 |
| FLJ39639 | uncharacterized protein FLJ39639 | 5.63E-10 | -1.54 |
| DAPK2 | death-associated protein kinase 2 | 3.88E-08 | -1.54 |
| EMR1 | egf-like module containing, mucin-like, hormone receptor-like 1 | 8.19E-04 | -1.54 |
| ADAM8 | ADAM metallopeptidase domain 8 | 5.08E-08 | -1.54 |
| RN5S343 | RNA, 5S ribosomal 343 | 5.57E-07 | -1.54 |
| STK40 | serine/threonine kinase 40 | 1.90E-10 | -1.54 |
| KLHL21 | kelch-like 21 (Drosophila) | 2.32E-13 | -1.54 |
| ARSA | arylsulfatase A | 2.96E-09 | -1.54 |
| FCGRT | Fc fragment of IgG, receptor, transporter, alpha | 1.12E-10 | -1.54 |
| EIF2C4 | eukaryotic translation initiation factor 2C, 4 | 1.22E-08 | -1.55 |
| STX3 | syntaxin 3 | 7.65E-08 | -1.55 |
| ANP32A-IT1 | ANP32A intronic transcript 1 (non-protein coding) | 7.77E-12 | -1.55 |
| SNORA45 | small nucleolar RNA, H/ACA box 45 | 1.21E-03 | -1.55 |
| PPTC7 | PTC7 protein phosphatase homolog (S. cerevisiae) | 1.62E-15 | -1.55 |
| SIRPB2 | signal-regulatory protein beta 2 | 9.89E-07 | -1.55 |
| RNF144B | ring finger protein 144B | 1.87E-06 | -1.55 |
| ERV3-1 | endogenous retrovirus group 3, member 1 | 1.61E-04 | -1.56 |
| TALDO1 | transaldolase 1 | 4.73E-09 | -1.56 |
| TKT | transketolase | 1.35E-09 | -1.56 |
| NOTCH1 | notch 1 | 3.17E-08 | -1.56 |
| ZNF852 | zinc finger protein 852 | 5.93E-07 | -1.56 |
| SLC6A6 | solute carrier family 6 (neurotransmitter transporter, taurine), member 6 | 3.13E-10 | -1.57 |
| P2RY14 | purinergic receptor P2Y, G-protein coupled, 14 | 1.13E-03 | -1.57 |
| FAM53C | family with sequence similarity 53, member C | 2.43E-10 | -1.57 |
| NR6A1 | nuclear receptor subfamily 6, group A, member 1 | 2.53E-14 | -1.57 |
| TUBA1A | tubulin, alpha 1a | 2.21E-07 | -1.57 |
| SNORA65 | small nucleolar RNA, H/ACA box 65 | 5.11E-06 | -1.57 |
| ARAP1 | ArfGAP with RhoGAP domain, ankyrin repeat and PH domain 1 | 2.56E-09 | -1.57 |
| TIMM23 | translocase of inner mitochondrial membrane 23 homolog (yeast) | 6.81E-09 | -1.58 |
| SCARNA10 | small Cajal body-specific RNA 10 | 2.37E-03 | -1.58 |
| PCMTD1 | protein-L-isoaspartate (D-aspartate) O-methyltransferase domain containing 1 | 7.87E-07 | -1.59 |
| SPDYE2 | speedy homolog E2 (Xenopus laevis) | 1.67E-07 | -1.59 |
| CD82 | CD82 molecule | 1.13E-11 | -1.60 |
| ENTPD1 | ectonucleoside triphosphate diphosphohydrolase 1 | 1.75E-08 | -1.60 |
| GLT1D1 | glycosyltransferase 1 domain containing 1 | 1.87E-06 | -1.60 |
| KIAA0226L | KIAA0226-like | 1.41E-05 | -1.60 |
| SNORA23 | small nucleolar RNA, H/ACA box 23 | 7.44E-03 | -1.60 |
| CRISPLD2 | cysteine-rich secretory protein LCCL domain containing 2 | 1.28E-04 | -1.61 |
| S1PR4 | sphingosine-1-phosphate receptor 4 | 1.69E-13 | -1.61 |
| WDR74 | WD repeat domain 74 | 2.21E-03 | -1.61 |
| MAGT1 | magnesium transporter 1 | 9.02E-08 | -1.62 |
| SNORA71A | small nucleolar RNA, H/ACA box 71A | 3.24E-03 | -1.62 |
| UFM1 | ubiquitin-fold modifier 1 | 6.17E-09 | -1.62 |
| PRRG4 | proline rich Gla (G-carboxyglutamic acid) 4 (transmembrane) | 3.66E-05 | -1.62 |
| LPCAT2 | lysophosphatidylcholine acyltransferase 2 | 3.14E-07 | -1.62 |
| C7orf53 | chromosome 7 open reading frame 53 | 2.09E-07 | -1.63 |
| ORM1 | orosomucoid 1 | 5.49E-05 | -1.63 |
| MME | membrane metallo-endopeptidase | 5.04E-04 | -1.64 |
| KY | kyphoscoliosis peptidase | 3.84E-07 | -1.64 |
| TMEM14E | transmembrane protein 14E | 4.23E-06 | -1.64 |
| SNORA46 | small nucleolar RNA, H/ACA box 46 | 1.80E-07 | -1.64 |
| TNFAIP2 | tumor necrosis factor, alpha-induced protein 2 | 5.97E-09 | -1.64 |
| KIAA0247 | KIAA0247 | 4.94E-12 | -1.65 |
| RN5S74 | RNA, 5S ribosomal 74 | 5.90E-04 | -1.65 |
| ST6GALNAC2 | ST6 (alpha-N-acetyl-neuraminyl-2,3-beta-galactosyl-1,3)-N-acetycetylgalactosaminide alpha-2,6-sialyltransferase 2 | 1.45E-06 | -1.65 |
| DLEU1 | deleted in lymphocytic leukemia 1 (non-protein coding) | 2.69E-09 | -1.65 |
| FRY | furry homolog (Drosophila) | 1.48E-09 | -1.65 |
| IMPA2 | inositol(myo)-1(or 4)-monophosphatase 2 | 3.23E-12 | -1.67 |
| EXTL3 | exostoses (multiple)-like 3 | 3.54E-10 | -1.67 |
| SNORD32B | small nucleolar RNA, C/D box 32B | 1.50E-06 | -1.67 |
| SNORA3 | small nucleolar RNA, H/ACA box 3 | 1.16E-03 | -1.67 |
| SNORA57 | small nucleolar RNA, H/ACA box 57 | 2.11E-03 | -1.68 |
| MMP9 | matrix metallopeptidase 9 | 2.31E-03 | -1.68 |
| FRAT1 | frequently rearranged in advanced T-cell lymphomas | 2.55E-10 | -1.70 |
| UGT2B7 | UDP glucuronosyltransferase 2 family, polypeptide B7 | 6.84E-07 | -1.70 |
| C20orf3 | chromosome 20 open reading frame 3 | 1.03E-12 | -1.70 |
| TPST1 | tyrosylprotein sulfotransferase 1 | 1.09E-06 | -1.70 |
| GAFA3 | FGF-2 activity-associated protein 3 | 8.70E-11 | -1.71 |
| KCNRG | potassium channel regulator | 4.94E-08 | -1.72 |
| STARD10-AS1 | STARD10 antisense RNA 1 (non-protein coding) | 1.04E-04 | -1.72 |
| RPL7A | ribosomal protein L7a | 2.55E-03 | -1.77 |
| BRK1 | BRICK1, SCAR/WAVE actin-nucleating complex subunit | 4.95E-06 | -1.78 |
| PDE4DIP | phosphodiesterase 4D interacting protein | 5.16E-08 | -1.78 |
| IFNGR1 | interferon gamma receptor 1 | 7.04E-08 | -1.79 |
| SNORA41 | small nucleolar RNA, H/ACA box 41 | 2.76E-06 | -1.79 |
| GAS5 | growth arrest-specific 5 (non-protein coding) | 1.12E-03 | -1.80 |
| WLS | wntless homolog (Drosophila) | 2.79E-04 | -1.81 |
| SNORA37 | small nucleolar RNA, H/ACA box 37 | 5.81E-08 | -1.84 |
| SNORA71C | small nucleolar RNA, H/ACA box 71C | 2.79E-07 | -1.89 |
| RNASEK | ribonuclease, RNase K | 6.73E-07 | -1.89 |
| RPL13A | ribosomal protein L13a | 1.30E-06 | -1.91 |
| NOL7 | nucleolar protein 7, 27kDa | 1.40E-07 | -1.92 |
| NCOR2 | nuclear receptor corepressor 2 | 1.86E-08 | -1.99 |
| RPS2 | ribosomal protein S2 | 1.43E-03 | -2.00 |
| ROCK1 | Rho-associated, coiled-coil containing protein kinase 1 | 1.40E-06 | -2.02 |
| SNORD56B | small nucleolar RNA, C/D box 56B | 5.39E-06 | -2.05 |
| SNORD3A | small nucleolar RNA, C/D box 3A | 1.32E-03 | -2.05 |
| SNORA70G | small nucleolar RNA, H/ACA box 70G | 1.23E-07 | -2.07 |
| VTRNA1-1 | vault RNA 1-1 | 5.61E-03 | -2.14 |
| IL5RA | interleukin 5 receptor, alpha | 4.97E-04 | -2.15 |
| SNORA42 | small nucleolar RNA, H/ACA box 42 | 8.97E-06 | -2.27 |
| ND6 | NADH dehydrogenase, subunit 6 (complex I) | 1.49E-04 | -2.29 |
| SDAD1 | SDA1 domain containing 1 | 3.67E-09 | -2.32 |
| TOB2 | transducer of ERBB2, 2 | 5.16E-06 | -2.35 |
| RAB7A | RAB7A, member RAS oncogene family | 3.17E-07 | -2.39 |
| GNG2 | guanine nucleotide binding protein (G protein), gamma 2 | 2.00E-09 | -2.68 |
| FYB | FYN binding protein | 5.29E-08 | -2.72 |
| MALAT1 | metastasis associated lung adenocarcinoma transcript 1 (non-protein coding) | 7.80E-07 | -2.82 |
| DNAJA2 | DnaJ (Hsp40) homolog, subfamily A, member 2 | 7.32E-11 | -3.18 |

**S5** Genes modulated by interferon-alpha at treatment week 4 specifically in patients who do not develop depression

| **Gene Symbol** | **Gene assignment** | **p value** | **Fold change** |
| --- | --- | --- | --- |
| MMP8 | matrix metallopeptidase 8 (neutrophil collagenase) | 4.56E-03 | 1.80 |
| CKS2 | CDC28 protein kinase regulatory subunit 2 | 6.37E-04 | 1.61 |
| HIST1H4D | histone cluster 1, H4d | 1.52E-06 | 1.60 |
| DHRS9 | dehydrogenase/reductase (SDR family) member 9 | 7.21E-05 | 1.58 |
| CMKLR1 | chemokine-like receptor 1 | 6.45E-04 | 1.53 |
| GPSM2 | G-protein signaling modulator 2 | 1.42E-04 | 1.51 |
| SCARNA9L | small Cajal body-specific RNA 9-like | 5.90E-05 | 1.48 |
| TTPAL | tocopherol (alpha) transfer protein-like | 6.57E-06 | 1.47 |
| C17orf109 | chromosome 17 open reading frame 109 | 3.19E-04 | 1.47 |
| FANCL | Fanconi anemia, complementation group L | 3.85E-08 | 1.46 |
| CYSLTR1 | cysteinyl leukotriene receptor 1 | 3.75E-07 | 1.45 |
| CD36 | CD36 molecule (thrombospondin receptor) | 1.42E-03 | 1.44 |
| SLFN12 | schlafen family member 12 | 2.57E-05 | 1.44 |
| SHISA5 | shisa homolog 5 (Xenopus laevis) | 6.48E-10 | 1.43 |
| STAT1 | signal transducer and activator of transcription 1, 91kDa | 2.53E-07 | 1.43 |
| RHCE | Rh blood group, CcEe antigens | 5.19E-05 | 1.43 |
| TXNDC16 | thioredoxin domain containing 16 | 7.84E-05 | 1.41 |
| C4orf33 | chromosome 4 open reading frame 33 | 7.13E-06 | 1.40 |
| TBC1D9 | TBC1 domain family, member 9 (with GRAM domain) | 2.41E-07 | -1.40 |
| BEST1 | bestrophin 1 | 7.10E-07 | -1.40 |
| CD1C | CD1c molecule | 3.09E-11 | -1.40 |
| DUSP22 | dual specificity phosphatase 22 | 3.20E-12 | -1.40 |
| SELP | selectin P (granule membrane protein 140kDa, antigen CD62) | 3.28E-04 | -1.40 |
| JAK3 | Janus kinase 3 | 1.45E-11 | -1.40 |
| LGALSL | lectin, galactoside-binding-like | 1.23E-03 | -1.41 |
| C6orf25 | chromosome 6 open reading frame 25 | 1.42E-03 | -1.41 |
| ITGB8 | integrin, beta 8 | 1.48E-11 | -1.41 |
| ARRB2 | arrestin, beta 2 | 1.17E-13 | -1.41 |
| B3GNT5 | UDP-GlcNAc:betaGal beta-1,3-N-acetylglucosaminyltransferase 5 | 5.63E-05 | -1.41 |
| LIN7A | lin-7 homolog A (C. elegans) | 3.60E-04 | -1.41 |
| CTDSPL | CTD (carboxy-terminal domain, RNA polymerase II, polypeptide A) small phosphatase-like | 1.03E-05 | -1.42 |
| SLC12A9 | solute carrier family 12 (potassium/chloride transporters), member 9 | 9.14E-12 | -1.42 |
| RTN1 | reticulon 1 | 3.87E-07 | -1.42 |
| HIPK3 | homeodomain interacting protein kinase 3 | 4.86E-10 | -1.42 |
| PAM | peptidylglycine alpha-amidating monooxygenase | 2.53E-06 | -1.43 |
| C15orf39 | chromosome 15 open reading frame 39 | 8.03E-09 | -1.43 |
| ITGB5 | integrin, beta 5 | 4.21E-04 | -1.43 |
| PPM1L | protein phosphatase, Mg2+/Mn2+ dependent, 1L | 1.15E-04 | -1.44 |
| HCAR3 | hydroxycarboxylic acid receptor 3 | 3.22E-03 | -1.45 |
| GSTM2 | glutathione S-transferase mu 2 (muscle) | 8.35E-04 | -1.45 |
| CSF2RB | colony stimulating factor 2 receptor, beta, low-affinity (granulocyte-macrophage) | 4.83E-07 | -1.45 |
| C15orf54 | chromosome 15 open reading frame 54 | 1.07E-06 | -1.46 |
| DNAJC25-GNG10 | DNAJC25-GNG10 readthrough | 5.53E-05 | -1.47 |
| CXCL16 | chemokine (C-X-C motif) ligand 16 | 6.92E-06 | -1.48 |
| BEND2 | BEN domain containing 2 | 5.56E-04 | -1.49 |
| BASP1 | brain abundant, membrane attached signal protein 1 | 1.83E-05 | -1.50 |
| TSPAN33 | tetraspanin 33 | 3.57E-06 | -1.50 |
| DNM3 | dynamin 3 | 3.01E-05 | -1.51 |
| JAM3 | junctional adhesion molecule 3 | 5.51E-08 | -1.53 |
| RAB27B | RAB27B, member RAS oncogene family | 1.15E-04 | -1.54 |
| SLC25A37 | solute carrier family 25 (mitochondrial iron transporter), member 37 | 1.16E-05 | -1.54 |
| GNAZ | guanine nucleotide binding protein (G protein), alpha z polypeptide | 2.14E-05 | -1.55 |
| FSTL1 | follistatin-like 1 | 5.19E-06 | -1.55 |
| FHL1 | four and a half LIM domains 1 | 5.50E-06 | -1.55 |
| LY6G6F | lymphocyte antigen 6 complex, locus G6F | 7.83E-05 | -1.57 |
| GUCY1B3 | guanylate cyclase 1, soluble, beta 3 | 4.91E-07 | -1.59 |
| MS4A3 | membrane-spanning 4-domains, subfamily A, member 3 (hematopoietic cell-specific) | 2.66E-03 | -1.62 |
| PDE5A | phosphodiesterase 5A, cGMP-specific | 2.11E-04 | -1.63 |
| F13A1 | coagulation factor XIII, A1 polypeptide | 8.85E-04 | -1.63 |
| SDPR | AK290378 // SDPR // serum deprivation response | 4.11E-05 | -1.64 |
| PF4 | platelet factor 4 | 5.03E-04 | -1.64 |
| PROS1 | protein S (alpha) | 7.62E-14 | -1.65 |
| ITGA2B | integrin, alpha 2b (platelet glycoprotein IIb of IIb/IIIa complex, antigen CD41) | 2.40E-04 | -1.69 |
| TREML1 | triggering receptor expressed on myeloid cells-like 1 | 2.94E-04 | -1.70 |
| NRGN | neurogranin (protein kinase C substrate, RC3) | 4.79E-05 | -1.70 |
| GP1BA | glycoprotein Ib (platelet), alpha polypeptide | 1.19E-05 | -1.78 |
| ELOVL7 | ELOVL fatty acid elongase 7 | 1.49E-05 | -1.80 |
| RN5S353 | RNA, 5S ribosomal 353 | 1.79E-03 | -1.82 |
| PRKAR2B | protein kinase, cAMP-dependent, regulatory, type II, beta | 2.68E-05 | -1.85 |
| ITGB3 | integrin, beta 3 (platelet glycoprotein IIIa, antigen CD61) | 1.35E-04 | -2.53 |

**S6** Genes modulated by interferon-alpha at treatment week 24 specifically in patients who develop depression

| **Gene symbol** | **Gene assignment** | **p value** | **Fold change** |
| --- | --- | --- | --- |
| HBZ | hemoglobin, zeta | 3.16E-03 | 3.23 |
| ANK1 | ankyrin 1, erythrocytic | 1.59E-04 | 1.92 |
| TSPAN7 | tetraspanin 7 | 8.08E-04 | 1.90 |
| NEDD4L | neural precursor cell expressed, developmentally down-regulated 4-ike, E3 ubiquitin protein ligase | 9.01E-05 | 1.90 |
| SEC14L4 | SEC14-like 4 (S. cerevisiae) | 1.34E-04 | 1.89 |
| NT5M | 5',3'-nucleotidase, mitochondrial | 3.87E-06 | 1.86 |
| AIF1 | allograft inflammatory factor 1 | 2.24E-05 | 1.78 |
| DNAJC15 | DnaJ (Hsp40) homolog, subfamily C, member 15 | 1.71E-07 | 1.74 |
| NFIA | nuclear factor I/A | 3.10E-06 | 1.73 |
| ATP5G1 | ATP synthase, H+ transporting, mitochondrial Fo complex, subunit C1 (subunit 9) | 7.33E-05 | 1.72 |
| RANBP10 | RAN binding protein 10 | 3.91E-04 | 1.71 |
| IGF2BP2 | insulin-like growth factor 2 mRNA binding protein 2 | 3.63E-03 | 1.70 |
| EPB49 | erythrocyte membrane protein band 4.9 (dematin) | 1.06E-04 | 1.69 |
| SWT1 | SWT1 RNA endoribonuclease homolog (S. cerevisiae) | 8.60E-08 | 1.68 |
| GAPT | GRB2-binding adaptor protein, transmembrane | 3.15E-08 | 1.68 |
| MARCH2 | membrane-associated ring finger (C3HC4) 2, E3 ubiquitin protein ligase | 7.06E-05 | 1.66 |
| KIF15 | kinesin family member 15 | 1.58E-06 | 1.66 |
| RFESD | Rieske (Fe-S) domain containing | 2.32E-07 | 1.66 |
| SNX2 | sorting nexin 2 | 8.47E-09 | 1.65 |
| LRRCC1 | leucine rich repeat and coiled-coil centrosomal protein 1 | 2.14E-10 | 1.64 |
| CD69 | CD69 molecule | 1.26E-06 | 1.64 |
| KLRF1 | killer cell lectin-like receptor subfamily F, member 1 | 8.63E-03 | 1.64 |
| TFEC | transcription factor EC | 6.38E-06 | 1.63 |
| CDKN3 | cyclin-dependent kinase inhibitor 3 | 9.08E-05 | 1.62 |
| CLEC4D | C-type lectin domain family 4, member D | 7.74E-04 | 1.62 |
| CLIC2 | chloride intracellular channel 2 | 8.96E-04 | 1.61 |
| RPL41 | ribosomal protein L41 | 4.93E-05 | 1.60 |
| RILP | Rab interacting lysosomal protein | 1.89E-03 | 1.59 |
| ALS2CR12 | amyotrophic lateral sclerosis 2 (juvenile) chromosome region, candidate 12 | 7.85E-06 | 1.58 |
| E2F2 | E2F transcription factor 2 | 3.22E-03 | 1.58 |
| ZNF675 | zinc finger protein 675 | 2.96E-04 | 1.58 |
| UBAC1 | UBA domain containing 1 | 1.77E-06 | 1.58 |
| THOC7 | THO complex 7 homolog (Drosophila) | 4.96E-08 | 1.58 |
| TBCEL | tubulin folding cofactor E-like | 1.73E-04 | 1.57 |
| FAM210B | family with sequence similarity 210, member B | 1.24E-03 | 1.57 |
| FMNL2 | formin-like 2 | 2.08E-07 | 1.57 |
| ANKRD36C | ankyrin repeat domain 36C | 2.53E-07 | 1.57 |
| ELOF1 | elongation factor 1 homolog (S. cerevisiae) | 3.54E-03 | 1.57 |
| IFI16 | interferon, gamma-inducible protein 16 | 3.13E-13 | 1.56 |
| CASC5 | cancer susceptibility candidate 5 | 4.56E-09 | 1.56 |
| TPMT | thiopurine S-methyltransferase | 5.49E-09 | 1.56 |
| PPME1 | protein phosphatase methylesterase 1 | 9.36E-08 | 1.55 |
| MAFB | v-maf musculoaponeurotic fibrosarcoma oncogene homolog B (avian) | 1.16E-07 | 1.54 |
| BRCA2 | breast cancer 2, early onset | 2.20E-09 | 1.54 |
| CD48 | CD48 molecule | 5.81E-06 | 1.54 |
| COMMD6 | COMM domain containing 6 | 2.06E-03 | 1.53 |
| XPO7 | exportin 7 | 6.33E-05 | 1.53 |
| DNAJA4 | DnaJ (Hsp40) homolog, subfamily A, member 4 | 4.62E-04 | 1.53 |
| RHBDD1 | rhomboid domain containing 1 | 5.60E-08 | 1.53 |
| TXNDC16 | thioredoxin domain containing 16 | 1.18E-05 | 1.52 |
| DNA2 | DNA replication helicase 2 homolog (yeast) | 7.14E-09 | 1.52 |
| SLIRP | SRA stem-loop interacting RNA binding protein | 3.62E-04 | 1.52 |
| GAB1 | GRB2-associated binding protein 1 | 2.87E-05 | 1.52 |
| ARHGAP42 | ho GTPase activating protein 42 | 6.26E-06 | 1.52 |
| RPL5 | ribosomal protein L5 | 1.94E-03 | 1.52 |
| GNA12 | guanine nucleotide binding protein (G protein) alpha 12 | 1.35E-03 | 1.52 |
| CCBP2 | chemokine binding protein 2 | 5.82E-05 | 1.52 |
| ZNF79 | zinc finger protein 79 | 1.92E-14 | 1.52 |
| ZWILCH | Zwilch, kinetochore associated, homolog (Drosophila) | 1.01E-07 | 1.51 |
| GBP3 | guanylate binding protein 3 | 5.88E-06 | 1.51 |
| ZNF496 | zinc finger protein 496 | 5.97E-11 | 1.51 |
| CCDC18 | coiled-coil domain containing 18 | 1.57E-06 | 1.51 |
| PDHX | pyruvate dehydrogenase complex, component X | 2.05E-10 | 1.51 |
| DLGAP5 | discs, large (Drosophila) homolog-associated protein 5 | 7.15E-06 | 1.50 |
| LPAR6 | lysophosphatidic acid receptor 6 | 1.31E-03 | 1.50 |
| NPRL3 | nitrogen permease regulator-like 3 (S. cerevisiae) | 1.25E-03 | 1.50 |
| TRIM10 | tripartite motif containing 10 | 2.85E-04 | 1.50 |
| ERH | enhancer of rudimentary homolog (Drosophila) | 1.04E-04 | 1.50 |
| RRM2 | ribonucleotide reductase M2 | 5.77E-12 | 1.50 |
| ZC3H6 | zinc finger CCCH-type containing 6 | 3.25E-11 | 1.50 |
| ATAD5 | ATPase family, AAA domain containing 5 | 2.61E-08 | 1.50 |
| BLZF1 | basic leucine zipper nuclear factor 1 | 2.66E-08 | 1.50 |
| HCST | hematopoietic cell signal transducer | 1.82E-04 | 1.49 |
| LIN9 | lin-9 homolog (C. elegans) | 3.86E-06 | 1.49 |
| PLVAP | plasmalemma vesicle associated protein | 7.06E-03 | 1.49 |
| FAM190A | family with sequence similarity 190, member A | 1.16E-04 | 1.49 |
| NUP62CL | nucleoporin 62kDa C-terminal like | 2.50E-07 | 1.49 |
| GPD2 | glycerol-3-phosphate dehydrogenase 2 (mitochondrial) | 4.28E-06 | 1.48 |
| ZNF844 | zinc finger protein 844 | 5.40E-05 | 1.48 |
| ZNF441 | zinc finger protein 441 | 2.21E-05 | 1.48 |
| TRIM38 | tripartite motif containing 38 | 3.47E-10 | 1.48 |
| BMP2K | BMP2 inducible kinase | 8.56E-07 | 1.48 |
| POMP | proteasome maturation protein | 2.03E-06 | 1.48 |
| TIMMDC1 | translocase of inner mitochondrial membrane domain containing 1 | 8.83E-07 | 1.48 |
| POLR2K | polymerase (RNA) II (DNA directed) polypeptide K, 7.0kDa | 1.24E-03 | 1.47 |
| MGST3 | microsomal glutathione S-transferase 3 | 1.22E-03 | 1.47 |
| LRRC37B | leucine rich repeat containing 37B | 1.04E-09 | 1.47 |
| COPS2 | COP9 constitutive photomorphogenic homolog subunit 2 (Arabidopsis) | 5.47E-05 | 1.47 |
| DCTN6 | dynactin 6 | 2.38E-06 | 1.46 |
| ZNF260 | zinc finger protein 260 | 2.21E-04 | 1.46 |
| SPECC1 | sperm antigen with calponin homology and coiled-coil domains 1 | 3.32E-05 | 1.46 |
| ATP5C1 | ATP synthase, H+ transporting, mitochondrial F1 complex, gamma polypeptide 1 | 8.35E-07 | 1.46 |
| ZCRB1 | zinc finger CCHC-type and RNA binding motif 1 | 5.91E-07 | 1.46 |
| MCM8 | minichromosome maintenance complex component 8 | 1.94E-05 | 1.46 |
| AKAP7 | A kinase (PRKA) anchor protein 7 | 5.32E-09 | 1.45 |
| SFT2D1 | FT2 domain containing 1 | 6.41E-07 | 1.45 |
| UBQLNL | ubiquilin-like | 4.78E-10 | 1.45 |
| PSMA4 | proteasome (prosome, macropain) subunit, alpha type, 4 | 2.02E-04 | 1.45 |
| KIF14 | kinesin family member 14 | 5.75E-06 | 1.45 |
| USP12 | ubiquitin specific peptidase 12 | 1.18E-03 | 1.45 |
| PLSCR4 | phospholipid scramblase 4 | 1.23E-07 | 1.45 |
| TNFSF10 | tumor necrosis factor (ligand) superfamily, member 10 | 5.56E-10 | 1.45 |
| ARV1 | ARV1 homolog (S. cerevisiae) | 1.07E-05 | 1.45 |
| NSA2 | NSA2 ribosome biogenesis homolog (S. cerevisiae) | 9.07E-05 | 1.45 |
| NUCB2 | nucleobindin 2 | 2.76E-06 | 1.45 |
| MERTK | c-mer proto-oncogene tyrosine kinase | 1.16E-04 | 1.45 |
| ABCB10 | ATP-binding cassette, sub-family B (MDR/TAP), member 10 | 1.51E-06 | 1.45 |
| ZNF480 | zinc finger protein 480 | 2.36E-04 | 1.44 |
| HINT2 | histidine triad nucleotide binding protein 2 | 2.69E-04 | 1.44 |
| CRAT | carnitine O-acetyltransferase | 6.07E-03 | 1.44 |
| GABPB2 | GA binding protein transcription factor, beta subunit 2 | 6.28E-09 | 1.44 |
| HSPB1 | heat shock 27kDa protein 1 | 7.82E-04 | 1.44 |
| IL18 | interleukin 18 (interferon-gamma-inducing factor) | 6.01E-05 | 1.44 |
| GPSM2 | G-protein signaling modulator 2 | 1.87E-03 | 1.44 |
| TPX2 | TPX2, microtubule-associated, homolog (Xenopus laevis) | 5.94E-09 | 1.44 |
| MRPS24 | mitochondrial ribosomal protein S24 | 1.95E-05 | 1.44 |
| NT5C3 | 5'-nucleotidase, cytosolic III | 3.49E-10 | 1.44 |
| QSER1 | glutamine and serine rich 1 | 1.10E-06 | 1.44 |
| BMPR2 | bone morphogenetic protein receptor, type II (serine/threonine kinase) | 7.28E-07 | 1.44 |
| SUB1 | SUB1 homolog (S. cerevisiae) | 3.69E-05 | 1.44 |
| NRIP1 | nuclear receptor interacting protein 1 | 9.56E-06 | 1.43 |
| CMAS | cytidine monophosphate N-acetylneuraminic acid synthetase | 2.64E-05 | 1.43 |
| ZFAND4 | zinc finger, AN1-type domain 4 | 6.88E-06 | 1.43 |
| C1orf31 | chromosome 1 open reading frame 31 | 2.25E-05 | 1.43 |
| SP140 | SP140 nuclear body protein | 2.08E-06 | 1.43 |
| MZT2B | mitotic spindle organizing protein 2B | 1.87E-05 | 1.43 |
| TRAK2 | trafficking protein, kinesin binding 2 | 1.17E-03 | 1.43 |
| NAIP | NLR family, apoptosis inhibitory protein | 4.04E-05 | 1.42 |
| UBAP2 | ubiquitin associated protein 2 | 3.65E-10 | 1.42 |
| GPR180 | G protein-coupled receptor 180 | 8.14E-06 | 1.42 |
| ASPM | asp (abnormal spindle) homolog, microcephaly associated | 2.00E-07 | 1.42 |
| MYBL2 | v-myb myeloblastosis viral oncogene homolog (avian)-like 2 | 9.77E-09 | 1.42 |
| CBWD5 | COBW domain containing 5 | 1.36E-04 | 1.42 |
| VAMP8 | vesicle-associated membrane protein 8 (endobrevin) | 9.79E-04 | 1.42 |
| ZNF92 | zinc finger protein 92 | 5.32E-06 | 1.42 |
| ANP32B | acidic (leucine-rich) nuclear phosphoprotein 32 family, member B | 3.14E-05 | 1.42 |
| SSB | Sjogren syndrome antigen B (autoantigen La) | 4.98E-08 | 1.42 |
| BRIP1 | BRCA1 interacting protein C-terminal helicase 1 | 2.27E-06 | 1.42 |
| GIMAP7 | GTPase, IMAP family member 7 | 1.70E-04 | 1.42 |
| KIAA1586 | KIAA1586 | 9.25E-04 | 1.42 |
| UBL5 | ubiquitin-like 5 | 1.51E-03 | 1.42 |
| APOBEC3B | apolipoprotein B mRNA editing enzyme, catalytic polypeptide-like 3B | 4.22E-07 | 1.42 |
| ORC3 | origin recognition complex, subunit 3 | 7.93E-07 | 1.42 |
| C1orf9 | chromosome 1 open reading frame 9 | 5.40E-08 | 1.42 |
| RANBP9 | RAN binding protein 9 | 8.26E-09 | 1.42 |
| DNAJB4 | DnaJ (Hsp40) homolog, subfamily B, member 4 | 2.04E-06 | 1.42 |
| GTPBP2 | GTP binding protein 2 | 1.14E-07 | 1.41 |
| FAM46A | family with sequence similarity 46, member A | 8.23E-06 | 1.41 |
| SEC62 | SEC62 homolog (S. cerevisiae) | 2.69E-05 | 1.41 |
| TRAPPC5 | trafficking protein particle complex 5 | 1.62E-04 | 1.41 |
| SHISA7 | shisa homolog 7 (Xenopus laevis) | 3.18E-04 | 1.41 |
| ADD2 | adducin 2 (beta) | 2.02E-03 | 1.41 |
| WDR67 | WD repeat domain 67 | 1.58E-07 | 1.41 |
| EZH2 | enhancer of zeste homolog 2 (Drosophila) | 7.71E-11 | 1.40 |
| CYSLTR1 | cysteinyl leukotriene receptor 1 | 1.65E-05 | 1.40 |
| RNF213 | ring finger protein 213 | 1.25E-05 | 1.40 |
| ADAL | adenosine deaminase-like | 4.93E-05 | 1.40 |
| TRAT1 | T cell receptor associated transmembrane adaptor 1 | 6.58E-03 | 1.40 |
| UQCR10 | ubiquinol-cytochrome c reductase, complex III subunit X | 6.94E-06 | 1.40 |
| AGPAT9 | 1-acylglycerol-3-phosphate O-acyltransferase 9 | 5.35E-04 | -1.40 |
| MAP3K3 | mitogen-activated protein kinase kinase kinase 3 | 8.97E-07 | -1.40 |
| ENTPD1 | ectonucleoside triphosphate diphosphohydrolase 1 | 1.75E-05 | -1.40 |
| LPPR2 | lipid phosphate phosphatase-related protein type 2 | 1.23E-05 | -1.40 |
| C7orf53 | chromosome 7 open reading frame 53 | 1.46E-04 | -1.40 |
| SNORD24 | small nucleolar RNA, C/D box 24 | 4.02E-03 | -1.41 |
| CCL4 | chemokine (C-C motif) ligand 4 | 9.47E-05 | -1.41 |
| MYO1F | myosin IF | 5.84E-07 | -1.41 |
| LPCAT2 | lysophosphatidylcholine acyltransferase 2 | 1.41E-04 | -1.41 |
| SLC15A3 | solute carrier family 15, member 3 | 1.23E-07 | -1.41 |
| C11orf2 | chromosome 11 open reading frame 2 | 8.51E-14 | -1.41 |
| RPA2 | replication protein A2, 32kDa | 6.01E-09 | -1.41 |
| ZBTB34 | zinc finger and BTB domain containing 34 | 7.54E-07 | -1.42 |
| SNORA38 | small nucleolar RNA, H/ACA box 38 | 5.58E-05 | -1.42 |
| RCN3 | reticulocalbin 3, EF-hand calcium binding domain | 7.95E-09 | -1.42 |
| SNORD83B | small nucleolar RNA, C/D box 83B | 9.94E-05 | -1.42 |
| EYS | eyes shut homolog (Drosophila) | 1.10E-04 | -1.43 |
| FRY | furry homolog (Drosophila) | 5.79E-06 | -1.43 |
| LYSMD1 | LysM, putative peptidoglycan-binding, domain containing 1 | 1.76E-07 | -1.43 |
| FCGRT | Fc fragment of IgG, receptor, transporter, alpha | 2.80E-08 | -1.43 |
| GATA2 | GATA binding protein 2 | 2.55E-05 | -1.43 |
| KRT23 | keratin 23 (histone deacetylase inducible) | 5.55E-03 | -1.43 |
| PPTC7 | PTC7 protein phosphatase homolog (S. cerevisiae) | 4.96E-12 | -1.43 |
| SNORD30 | small nucleolar RNA, C/D box 30 | 1.10E-04 | -1.43 |
| NR6A1 | nuclear receptor subfamily 6, group A, member 1 | 1.15E-10 | -1.43 |
| FLJ39639 | uncharacterized protein FLJ39639 | 6.38E-08 | -1.43 |
| LSP1 | lymphocyte-specific protein 1 | 1.02E-04 | -1.43 |
| C14orf43 | chromosome 14 open reading frame 43 | 1.06E-07 | -1.43 |
| TKT | transketolase | 2.17E-07 | -1.44 |
| PID1 | phosphotyrosine interaction domain containing 1 | 6.76E-09 | -1.44 |
| IDS | iduronate 2-sulfatase | 2.27E-12 | -1.44 |
| TRIM13 | tripartite motif containing 13 | 3.23E-08 | -1.44 |
| CRISPLD2 | cysteine-rich secretory protein LCCL domain containing 2 | 2.19E-03 | -1.44 |
| ADAM8 | ADAM metallopeptidase domain 8 | 1.47E-06 | -1.44 |
| SNORA15 | small nucleolar RNA, H/ACA box 15 | 2.74E-08 | -1.44 |
| STK40 | serine/threonine kinase 40 | 1.42E-08 | -1.45 |
| EEF2 | eukaryotic translation elongation factor 2 | 1.50E-15 | -1.45 |
| KRTAP10-9 | keratin associated protein 10-9 | 1.05E-06 | -1.45 |
| KLHL21 | kelch-like 21 (Drosophila) | 4.11E-11 | -1.45 |
| ZNF185 | zinc finger protein 185 (LIM domain) | 2.52E-03 | -1.45 |
| ARHGAP9 | Rho GTPase activating protein 9 | 7.91E-10 | -1.45 |
| TNFAIP2 | tumor necrosis factor, alpha-induced protein 2 | 3.75E-06 | -1.45 |
| UGT2B7 | UDP glucuronosyltransferase 2 family, polypeptide B7 | 2.30E-04 | -1.45 |
| KCNJ2 | potassium inwardly-rectifying channel, subfamily J, member 2 | 1.33E-03 | -1.46 |
| ZNF587 | zinc finger protein 587 | 1.70E-06 | -1.46 |
| GAGE10 | G antigen 10 | 7.73E-04 | -1.46 |
| NOTCH1 | notch 1 | 1.16E-06 | -1.46 |
| SPDYE1 | speedy homolog E1 (Xenopus laevis) | 3.04E-09 | -1.46 |
| S1PR4 | sphingosine-1-phosphate receptor 4 | 4.53E-10 | -1.46 |
| PZP | pregnancy-zone protein | 1.77E-08 | -1.46 |
| SNORA74A | small nucleolar RNA, H/ACA box 74A | 1.64E-04 | -1.46 |
| ARAP1 | ArfGAP with RhoGAP domain, ankyrin repeat and PH domain 1 | 1.94E-07 | -1.46 |
| DUX4L7 | double homeobox 4 like 7 | 8.90E-04 | -1.46 |
| FRAT1 | frequently rearranged in advanced T-cell lymphomas | 1.34E-06 | -1.46 |
| SNORA45 | small nucleolar RNA, H/ACA box 45 | 3.44E-03 | -1.47 |
| EIF2C4 | eukaryotic translation initiation factor 2C, 4 | 1.27E-07 | -1.48 |
| PPP1R8 | protein phosphatase 1, regulatory subunit 8 | 1.91E-06 | -1.48 |
| TUBA1A | tubulin, alpha 1a | 3.23E-06 | -1.48 |
| ALDH2 | aldehyde dehydrogenase 2 family (mitochondrial) | 2.52E-04 | -1.48 |
| RNF144B | ring finger protein 144B | 4.04E-06 | -1.48 |
| LINC00216 | long intergenic non-protein coding RNA 216 | 3.78E-08 | -1.48 |
| GZMH | granzyme H (cathepsin G-like 2, protein h-CCPX) | 3.74E-04 | -1.49 |
| HIATL1 | hippocampus abundant transcript-like 1 | 4.09E-07 | -1.49 |
| PRR5L | proline rich 5 like | 2.66E-06 | -1.49 |
| GAFA2 | FGF-2 activity-associated protein 2 | 2.45E-05 | -1.49 |
| EIF4A1 | eukaryotic translation initiation factor 4A1 | 1.15E-03 | -1.49 |
| RLIM | ring finger protein, LIM domain interacting | 2.43E-06 | -1.50 |
| SLC11A1 | solute carrier family 11 (proton-coupled divalent metal ion transporter), member 1 | 2.17E-05 | -1.51 |
| DYSF | dysferlin, limb girdle muscular dystrophy 2B | 2.63E-04 | -1.51 |
| EXTL3 | exostoses (multiple)-like 3 | 9.93E-08 | -1.51 |
| RPPH1 | ribonuclease P RNA component H1 | 3.86E-03 | -1.51 |
| TSSK3 | testis-specific serine kinase 3 | 1.19E-07 | -1.51 |
| KY | kyphoscoliosis peptidase | 8.82E-06 | -1.52 |
| RNU6-79 | RNA, U6 small nuclear 79 | 1.18E-10 | -1.52 |
| HCRP1 | hepatocellular carcinoma-related HCRP1 | 7.86E-09 | -1.52 |
| SNORD94 | small nucleolar RNA, C/D box 94 | 5.92E-04 | -1.53 |
| SCARNA6 | small Cajal body-specific RNA 6 | 1.59E-03 | -1.53 |
| ZNF852 | zinc finger protein 852 | 8.54E-07 | -1.53 |
| TALDO1 | transaldolase 1 | 4.93E-09 | -1.54 |
| SCARNA10 | small Cajal body-specific RNA 10 | 3.21E-03 | -1.54 |
| KLRG1 | killer cell lectin-like receptor subfamily G, member 1 | 6.37E-04 | -1.55 |
| IMPA2 | inositol(myo)-1(or 4)-monophosphatase 2 | 2.65E-10 | -1.56 |
| SNORD105 | small nucleolar RNA, C/D box 105 | 1.15E-04 | -1.56 |
| UFM1 | ubiquitin-fold modifier 1 | 3.39E-08 | -1.56 |
| OLFM4 | olfactomedin 4 | 9.81E-04 | -1.56 |
| ZDHHC18 | zinc finger, DHHC-type containing 18 | 1.19E-08 | -1.57 |
| SNORA65 | small nucleolar RNA, H/ACA box 65 | 3.19E-06 | -1.57 |
| A2M | alpha-2-macroglobulin | 1.53E-08 | -1.58 |
| PCMTD1 | protein-L-isoaspartate (D-aspartate) O-methyltransferase domain containing 1 | 4.08E-07 | -1.59 |
| GAFA3 | FGF-2 activity-associated protein 3 | 5.18E-09 | -1.59 |
| RN5S343 | RNA, 5S ribosomal 343 | 4.04E-08 | -1.60 |
| TRGV1 | T cell receptor gamma variable 1 (non-functional) | 4.83E-03 | -1.60 |
| ARSA | arylsulfatase A | 7.29E-11 | -1.60 |
| SNORA46 | small nucleolar RNA, H/ACA box 46 | 3.04E-07 | -1.61 |
| MAGT1 | magnesium transporter 1 | 5.49E-08 | -1.61 |
| DNAJC8 | DnaJ (Hsp40) homolog, subfamily C, member 8 | 8.51E-10 | -1.62 |
| GPR56 | G protein-coupled receptor 56 | 6.07E-04 | -1.63 |
| STOM | stomatin | 4.45E-04 | -1.64 |
| DLEU1 | deleted in lymphocytic leukemia 1 (non-protein coding) | 1.26E-09 | -1.65 |
| ORM1 | orosomucoid 1 | 1.75E-05 | -1.67 |
| RN5S52 | RNA, 5S ribosomal 52 | 6.06E-03 | -1.68 |
| PDE4DIP | phosphodiesterase 4D interacting protein | 3.38E-07 | -1.69 |
| SPDYE2 | speedy homolog E2 (Xenopus laevis) | 2.35E-09 | -1.69 |
| RPL7A | ribosomal protein L7a | 4.15E-03 | -1.70 |
| SNORA71C | small nucleolar RNA, H/ACA box 71C | 8.97E-06 | -1.70 |
| SNORA52 | small nucleolar RNA, H/ACA box 52 | 8.77E-05 | -1.70 |
| TMEM14E | transmembrane protein 14E | 2.78E-07 | -1.72 |
| SNORD32B | small nucleolar RNA, C/D box 32B | 1.54E-07 | -1.73 |
| BRK1 | BRICK1, SCAR/WAVE actin-nucleating complex subunit | 6.50E-06 | -1.74 |
| MMP9 | matrix metallopeptidase 9 | 7.82E-04 | -1.75 |
| GAS5 | growth arrest-specific 5 (non-protein coding) | 1.42E-03 | -1.75 |
| KCNRG | potassium channel regulator | 2.12E-09 | -1.80 |
| SNORA41 | small nucleolar RNA, H/ACA box 41 | 1.31E-06 | -1.80 |
| IFNGR1 | interferon gamma receptor 1 | 2.57E-08 | -1.80 |
| RNASEK | ribonuclease, RNase K | 1.58E-06 | -1.82 |
| RN5S74 | RNA, 5S ribosomal 74 | 9.90E-06 | -1.89 |
| ROCK1 | Rho-associated, coiled-coil containing protein kinase 1 | 4.80E-06 | -1.91 |
| RPL13A | ribosomal protein L13a | 4.38E-07 | -1.94 |
| NOL7 | nucleolar protein 7, 27kDa | 9.76E-09 | -2.02 |
| SNORA70G | small nucleolar RNA, H/ACA box 70G | 9.33E-08 | -2.05 |
| VTRNA1-1 | vault RNA 1-1 | 6.84E-03 | -2.06 |
| SNORA42 | small nucleolar RNA, H/ACA box 42 | 4.05E-06 | -2.29 |
| SNORD3A | small nucleolar RNA, C/D box 3A | 9.13E-05 | -2.37 |
| ND6 | NADH dehydrogenase, subunit 6 (complex I) | 4.56E-05 | -2.40 |
| GNG2 | guanine nucleotide binding protein (G protein), gamma 2 | 2.45E-10 | -2.78 |
| MALAT1 | metastasis associated lung adenocarcinoma transcript 1 (non-protein coding) | 5.97E-07 | -2.79 |

**S7** Genes modulated by interferon-alpha at treatment week 24 specifically in patients who do not develop depression

| **Gene symbol** | **Gene assignment** | **p value** | **Fold change** |
| --- | --- | --- | --- |
| THEM5 | thioesterase superfamily member 5 | 8.53E-06 | 2.08 |
| AHSP | alpha hemoglobin stabilizing protein | 5.57E-04 | 1.87 |
| FADS2 | fatty acid desaturase 2 | 7.77E-05 | 1.81 |
| TSTA3 | tissue specific transplantation antigen P35B | 9.56E-04 | 1.72 |
| SNORD11 | small nucleolar RNA, C/D box 116 cluster | 3.95E-03 | 1.65 |
| HIST1H4D | histone cluster 1, H4d | 1.16E-07 | 1.64 |
| C3AR1 | complement component 3a receptor 1 | 3.48E-04 | 1.63 |
| HIST2H2BF | histone cluster 2, H2bf | 5.64E-07 | 1.59 |
| HBD | hemoglobin, delta | 3.77E-05 | 1.57 |
| PAQR9 | progestin and adipoQ receptor family member IX | 3.14E-08 | 1.55 |
| RPH3A | rabphilin 3A homolog (mouse) | 6.07E-10 | 1.55 |
| HIST1H2BH | histone cluster 1, H2bh | 4.07E-10 | 1.51 |
| STAT1 | signal transducer and activator of transcription 1, 91kDa | 8.47E-10 | 1.51 |
| LIMD1-AS1 | LIMD1 antisense RNA 1 (non-protein coding) | 1.09E-05 | 1.51 |
| HIST1H3F | histone cluster 1, H3f | 4.34E-07 | 1.51 |
| LGALS2 | lectin, galactoside-binding, soluble, 2 | 3.69E-03 | 1.50 |
| MT1G | metallothionein 1G | 1.15E-13 | 1.49 |
| FANCL | Fanconi anemia, complementation group L | 2.45E-09 | 1.48 |
| MT1E | metallothionein 1E | 1.22E-11 | 1.48 |
| H19 | H19, imprinted maternally expressed transcript (non-protein coding) | 6.40E-12 | 1.47 |
| SLC7A5 | solute carrier family 7 (amino acid transporter light chain, L system), member 5 | 3.64E-03 | 1.46 |
| AIM2 | absent in melanoma 2 | 1.19E-03 | 1.45 |
| LGALS1 | lectin, galactoside-binding, soluble, 1 | 3.63E-08 | 1.45 |
| AURKA | aurora kinase A | 2.53E-08 | 1.43 |
| FFAR3 | free fatty acid receptor 3 | 3.80E-05 | 1.43 |
| SLFN13 | schlafen family member 13 | 7.14E-06 | 1.43 |
| TYMP | thymidine phosphorylase | 3.36E-09 | 1.43 |
| LILRB4 | leukocyte immunoglobulin-like receptor, subfamily B (with TM and ITIM domains), member 4 | 2.44E-06 | 1.43 |
| CD300E | CD300e molecule | 9.79E-07 | 1.42 |
| SLC6A19 | solute carrier family 6 (neutral amino acid transporter), member 19 | 7.14E-03 | 1.42 |
| FAM70A | family with sequence similarity 70, member A | 5.45E-08 | 1.42 |
| SHISA5 | shisa homolog 5 (Xenopus laevis) | 3.02E-10 | 1.41 |
| RN5S399 | RNA, 5S ribosomal 399 | 1.23E-06 | 1.41 |
| XCL2 | chemokine (C motif) ligand 2 | 6.88E-03 | 1.41 |
| MT1H | metallothionein 1H | 6.46E-10 | 1.41 |
| SPIC | Spi-C transcription factor (Spi-1/PU.1 related) | 1.27E-05 | 1.40 |
| PPP3CA | protein phosphatase 3, catalytic subunit, alpha isozyme | 2.61E-14 | -1.40 |
| LIN7A | lin-7 homolog A (C. elegans) | 2.17E-04 | -1.40 |
| CYSLTR2 | cysteinyl leukotriene receptor 2 | 2.27E-03 | -1.40 |
| FAM129C | family with sequence similarity 129, member C | 2.29E-04 | -1.40 |
| BRI3BP | BRI3 binding protein | 2.54E-09 | -1.40 |
| THBD | thrombomodulin | 4.53E-08 | -1.40 |
| AOC2 | amine oxidase, copper containing 2 (retina-specific) | 6.70E-07 | -1.41 |
| SLC8A1 | solute carrier family 8 (sodium/calcium exchanger), member 1 | 6.08E-06 | -1.41 |
| MS4A1 | membrane-spanning 4-domains, subfamily A, member 1 | 3.87E-03 | -1.41 |
| PCSK6 | proprotein convertase subtilisin/kexin type 6 | 1.40E-07 | -1.41 |
| SSH2 | slingshot homolog 2 (Drosophila) | 1.11E-09 | -1.41 |
| CD1C | CD1c molecule | 1.39E-12 | -1.41 |
| HEATR5A | HEAT repeat containing 5A | 1.09E-10 | -1.41 |
| EHD3 | EH-domain containing 3 | 6.00E-05 | -1.41 |
| CSGALNACT1 | chondroitin sulfate N-acetylgalactosaminyltransferase 1 | 1.19E-05 | -1.41 |
| PTAFR | platelet-activating factor receptor | 7.42E-11 | -1.42 |
| RAPGEF2 | Rap guanine nucleotide exchange factor (GEF) 2 | 1.22E-08 | -1.42 |
| ABHD5 | abhydrolase domain containing 5 | 5.35E-07 | -1.42 |
| RPGR | retinitis pigmentosa GTPase regulator | 3.62E-12 | -1.42 |
| AFF3 | AF4/FMR2 family, member 3 | 6.25E-04 | -1.43 |
| ARHGAP24 | Rho GTPase activating protein 24 | 1.72E-07 | -1.43 |
| ASAP2 | ArfGAP with SH3 domain, ankyrin repeat and PH domain 2 | 2.72E-07 | -1.43 |
| ZNF860 | zinc finger protein 860 | 4.30E-03 | -1.43 |
| PLEKHG1 | pleckstrin homology domain containing, family G (with RhoGef dmain), member 1 | 1.60E-04 | -1.43 |
| MSL1 | male-specific lethal 1 homolog (Drosophila) | 2.16E-09 | -1.44 |
| FCRLA | Fc receptor-like A | 1.28E-04 | -1.44 |
| LY75 | lymphocyte antigen 75 | 6.36E-08 | -1.44 |
| RAB11FIP1 | RAB11 family interacting protein 1 (class I) | 2.98E-09 | -1.44 |
| IGHV3-72 | immunoglobulin heavy variable 3-72 | 5.17E-05 | -1.44 |
| ALCAM | activated leukocyte cell adhesion molecule | 8.14E-07 | -1.44 |
| SPATA6 | spermatogenesis associated 6 | 2.61E-09 | -1.44 |
| C6orf25 | chromosome 6 open reading frame 25 | 5.41E-04 | -1.44 |
| IGLJ3 | immunoglobulin lambda joining 3 | 5.42E-04 | -1.45 |
| ESYT1 | extended synaptotagmin-like protein 1 | 5.02E-07 | -1.45 |
| IGHV3-38 | immunoglobulin heavy variable 3-38 (non-functional) | 3.21E-04 | -1.45 |
| BASP1 | brain abundant, membrane attached signal protein 1 | 2.55E-05 | -1.45 |
| CTTN | cortactin | 2.40E-06 | -1.46 |
| WDFY4 | WDFY family member 4 | 8.80E-07 | -1.46 |
| PDLIM1 | PDZ and LIM domain 1 | 1.94E-06 | -1.46 |
| HIPK3 | homeodomain interacting protein kinase 3 | 4.43E-12 | -1.46 |
| RTN1 | reticulon 1 | 8.73E-09 | -1.46 |
| SELP | selectin P (granule membrane protein 140kDa, antigen CD62) | 2.31E-05 | -1.46 |
| CD24 | CD24 molecule | 9.86E-04 | -1.47 |
| MAK | male germ cell-associated kinase | 1.38E-04 | -1.48 |
| TRPM6 | transient receptor potential cation channel, subfamily M, member 6 | 4.05E-07 | -1.48 |
| ERV3-1 | endogenous retrovirus group 3, member 1 | 6.35E-05 | -1.49 |
| IL13RA1 | interleukin 13 receptor, alpha 1 | 1.83E-06 | -1.49 |
| CD79A | CD79a molecule, immunoglobulin-associated alpha | 3.98E-04 | -1.50 |
| ITGB5 | integrin, beta 5 | 2.85E-05 | -1.50 |
| RN5S164 | RNA, 5S ribosomal 164 | 2.78E-03 | -1.51 |
| LGALSL | lectin, galactoside-binding-like | 4.11E-05 | -1.51 |
| MS4A2 | membrane-spanning 4-domains, subfamily A, member 2 | 1.49E-05 | -1.52 |
| PROS1 | protein S (alpha) | 1.04E-11 | -1.52 |
| CTDSPL | CTD (carboxy-terminal domain, RNA polymerase II, polypeptide A) small phosphatase-like | 3.42E-08 | -1.52 |
| PTGS1 | prostaglandin-endoperoxide synthase 1 (prostaglandin G/H synthase and cyclooxygenase) | 8.23E-04 | -1.53 |
| MFAP3L | MFAP3L | 5.85E-08 | -1.54 |
| CD22 | CD22 molecule | 2.51E-03 | -1.54 |
| TSPAN33 | tetraspanin 33 | 1.70E-07 | -1.55 |
| JAM3 | junctional adhesion molecule 3 | 2.26E-09 | -1.57 |
| TBC1D9 | TBC1 domain family, member 9 (with GRAM domain) | 4.10E-12 | -1.57 |
| GNG11 | guanine nucleotide binding protein (G protein), gamma 11 | 2.34E-03 | -1.59 |
| FCRL1 | Fc receptor-like 1 | 2.01E-03 | -1.59 |
| P2RX5 | purinergic receptor P2X, ligand-gated ion channel, 5 | 3.64E-07 | -1.59 |
| RAB27B | RAB27B, member RAS oncogene family | 8.89E-06 | -1.60 |
| GUCY1B3 | guanylate cyclase 1, soluble, beta 3 | 7.99E-08 | -1.60 |
| GRAMD1C | GRAM domain containing 1C | 9.18E-08 | -1.60 |
| FHL1 | four and a half LIM domains 1 | 3.03E-07 | -1.61 |
| SHISA4 | shisa homolog 4 (Xenopus laevis) | 6.53E-05 | -1.61 |
| GNAZ | guanine nucleotide binding protein (G protein), alpha z polypeptide | 6.94E-07 | -1.63 |
| TREML1 | triggering receptor expressed on myeloid cells-like 1 | 3.37E-04 | -1.64 |
| BANK1 | B-cell scaffold protein with ankyrin repeats 1 | 4.36E-05 | -1.67 |
| C15orf54 | chromosome 15 open reading frame 54 | 1.35E-11 | -1.68 |
| FCRL2 | Fc receptor-like 2 | 2.26E-06 | -1.68 |
| BEND2 | BEN domain containing 2 | 2.89E-06 | -1.69 |
| OSBPL10 | oxysterol binding protein-like 10 | 7.62E-07 | -1.70 |
| TTI2 | TELO2 interacting protein 2 | 4.22E-03 | -1.72 |
| SPARC | secreted protein, acidic, cysteine-rich (osteonectin) | 1.85E-03 | -1.74 |
| DNM3 | dynamin 3 | 7.83E-09 | -1.75 |
| SCARNA4 | small Cajal body-specific RNA 4 | 5.58E-03 | -1.76 |
| RN5S353 | RNA, 5S ribosomal 353 | 1.71E-03 | -1.76 |
| RALGPS2 | Ral GEF with PH domain and SH3 binding motif 2 | 3.39E-08 | -1.77 |
| MS4A3 | membrane-spanning 4-domains, subfamily A, member 3 (hematopoietic cell-specific) | 1.08E-05 | -1.98 |
| IL5RA | interleukin 5 receptor, alpha | 1.89E-04 | -2.01 |
| THBS1 | thrombospondin 1 | 2.50E-06 | -2.16 |
| HLA-DQB1 | major histocompatibility complex, class II, DQ beta 1 | 1.17E-03 | -2.20 |

**S8** Pathways differentially modulated at treatment week 24 specifically in patients who develop depression (p≤0.05)

| **Pathway** | **Molecules** |
| --- | --- |
| Adenosine Nucleotides Degradation II | ADAL, NT5C3A, NT5M |
| Purine Nucleotides Degradation II (Aerobic) | ADAL, NT5C3A, NT5M |
| Pentose Phosphate Pathway (Non-oxidative Branch) | TKT, TALDO1 |
| Pentose Phosphate Pathway | TKT, TALDO1 |
| Guanosine Nucleotides Degradation III | NT5C3A, NT5M |
| Urate Biosynthesis/Inosine 5'-phosphate Degradation | NT5C3A, NT5M |
| Glutathione Redox Reactions I | CLIC2, MGST3 |
| EIF2 Signalling | RPL5, RPL7A, RPL13A, EIF4A1, RPL41, AGO4 |
| Death Receptor Signalling | HSPB1, NAIP, TNFSF10, ROCK1 |
| SAPK/JNK Signalling | GNA12, GNG2, GAB1, MAP3K3 |
| Protein Ubiquitination Pathway | HSPB1, DNAJC8, PSMA4, USP12, DNAJC15, DNAJB4, NEDD4L |
| Diphthamide Biosynthesis | EEF2 |
| Glycerol-3-phosphate Shuttle | GPD2 |
| Pancreatic Adenocarcinoma Signalling | NOTCH1, MMP9, E2F2, BRCA2 |
| NAD Salvage Pathway II | NT5C3A, NT5M |
| ERK5 Signalling | GNA12, GAB1, MAP3K3 |
| Cell Cycle Control of Chromosomal Replication | RPA2, ORC3 |
| Phenylethylamine Degradation I | ALDH2 |
| Myo-inositol Biosynthesis | IMPA2 |
| Mitochondrial Dysfunction | ATP5G1, UQCR10, ATP5C1, MT-ND6, GPD2 |


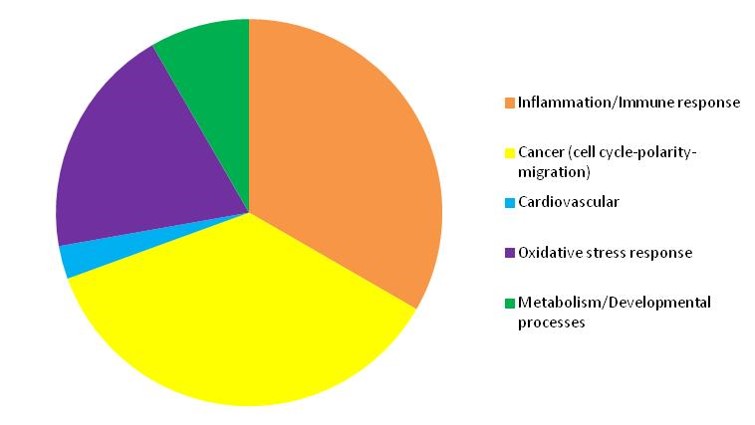


**S9** Role classification of pathways differentially modulated at treatment week 24 specifically in patients who develop depression
